# Complexed hyaluronic acid-based nanoparticles in cancer therapy and diagnosis: Research trends by natural language processing

**DOI:** 10.1016/j.heliyon.2024.e41246

**Published:** 2024-12-18

**Authors:** Abd Kakhar Umar, Patanachai K. Limpikirati, Bachtiar Rivai, Ilham Ardiansah, Sriwidodo Sriwidodo, Jittima Amie Luckanagul

**Affiliations:** a Pharmaceutical Sciences and Technology Program, Faculty of Pharmaceutical Sciences, Chulalongkorn University, Bangkok, 10330, Thailand; b Department of Food and Pharmaceutical Chemistry, Faculty of Pharmaceutical Sciences, Chulalongkorn University, Bangkok, 10330, Thailand; c Department of Pharmaceutics and Industrial Pharmacy, Faculty of Pharmaceutical Sciences, Chulalongkorn University, Bangkok, 10330, Thailand; d Department of Pharmaceutics and Pharmaceutical Technology, Faculty of Pharmacy, Universitas Padjadjaran, Sumedang, 45363, Indonesia; e Metabolomics for Life Sciences Research Unit, Chulalongkorn University, Bangkok, 10330, Thailand; f Department of Animal Husbandry, Faculty Veterinary Science, Chulalongkorn University, Bangkok, 10330, Thailand; g Medical Informatics Laboratory, ETFLIN, Palu City, 94225, Indonesia; h Center of Excellence in Plant-produced Pharmaceuticals, Chulalongkorn University, Bangkok, 10330, Thailand

**Keywords:** Latent dirichlet allocation, Named entity recognition, Surface conjugation, Multiple compartment system, Tumor microenvironment, Cancer dual targeting therapy

## Abstract

Hyaluronic acid (HA) is a popular surface modifier in targeted cancer delivery due to its receptor-binding abilities. However, HA alone faces limitations in lipid solubility, biocompatibility, and cell internalization, making it less effective as a standalone delivery system. This comprehensive study aimed to explore a dynamic landscape of complexation in HA-based nanoparticles in cancer therapy, examining diverse aspects from influential modifiers to emerging trends in cancer diagnostics. We discovered that certain active substances, such as 5-aminolevulinic acid, adamantane, and protamine, have been on trend in terms of their usage over the past decade. Dextran, streptavidin, and catechol emerge as intriguing conjugates for HA, coupled with nanostar, quantum dots, and nanoprobe structures for optimal drug delivery and diagnostics. Strategies like hypoxic conditioning, dual responsiveness, and pulse laser activation enhance controlled release, targeted delivery, and real-time diagnostic techniques like ultrasound imaging and X-ray computed tomography (X-ray CT). Based on our findings, conventional bibliometric tools fail to highlight relevant topics in this area, instead producing merely abstract and broad-meaning keywords. Extraction using Named Entity Recognition and topic search with Latent Dirichlet Allocation successfully revealed five representative topics with the ability to exclude irrelevant keywords. A shift in research focuses from optimizing chemical toxicity to particular targeting tactics and precise release mechanisms is evident. These findings reflect the dynamic landscape of HA-based nanoparticle research in cancer therapy, emphasizing advancements in targeted drug delivery, therapeutic efficacy, and multimodal diagnostic approaches to improve overall patient outcomes.

## Graphical abstract

**Figure:**
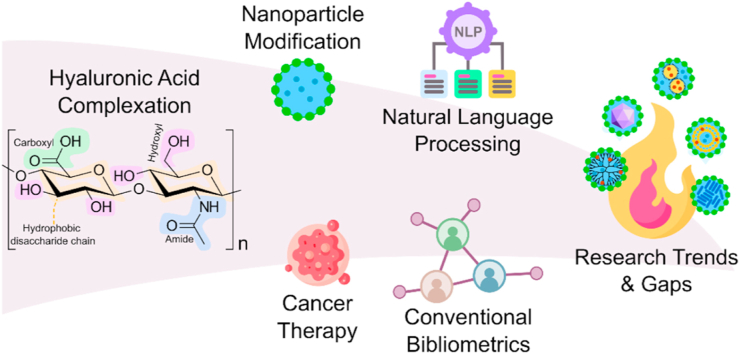

## 1 Introduction

**Fig. 1:**
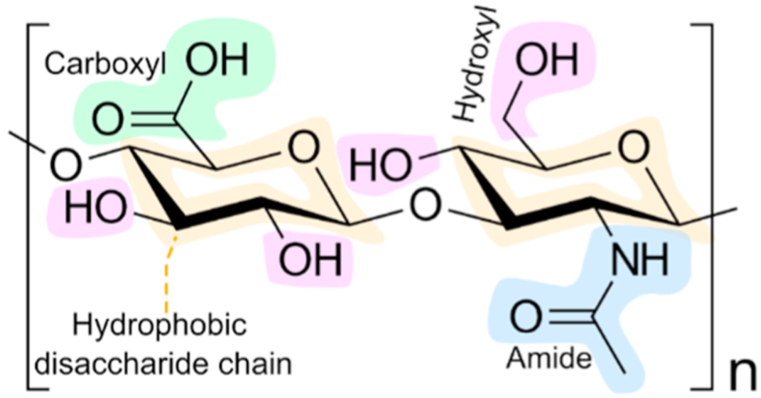
Hyaluronic acid structural features.

Cancer remains a leading cause of death, owing to a complex interplay of factors such as genetics, environment, lifestyle, and other variables [1,2]. Cancer patients are frequently treated with broad-spectrum medicines such as doxorubicin (DOX) [3] and paclitaxel (PTX) [4], but the issue is their selectivity [5,6]. Administering anticancer drugs via parenteral or indirect routes may result in systemic toxicity or adversely affect local healthy tissues [7]. As a result, targeted drug delivery has emerged as a widely explored topic, with one of the promising strategies utilizing hyaluronic acid (HA) (see Fig. 1). HA acts as a ligand for several cell surface receptors, making it a favorable surface modifier capable of effectively targeting CD44 receptors, receptors for hyaluronan-mediated motility (RHAMM or CD168), lymphatic vessel endothelial hyaluronan receptor-1 (LYVE-1), and stabilin receptors [[8], [9], [10], [11]]. Beyond cell surface receptors, HA also targets hyaluronidase enzymes, tumor microenvironment, macrophages, and immune cells, making it applicable to a broad spectrum of cancer therapies [[12], [13], [14], [15]]. However, HA may be insufficient as a standalone delivery system for drugs facing challenges such as lipid solubility [16], biocompatibility [17], resistance [18], and cell internalization [19]. Multi-drug delivery, aimed at reducing drug dose and systemic toxicity, poses challenges when relying solely on HA [[20], [21], [22]]. Moreover, dual-targeting and dual-responsive therapies require multi-compartments and/or sequential steps of drug release, necessitating a more complex HA system [23,24].

Complexation of HA is intriguing and useful. Carboxyl and hydroxyl groups in HA molecules facilitate this process, providing multiple opportunities for chemical alteration [25]. It also has a dominating hydrophilic region on its surface and a hydrophobic region on its disaccharide chain, which can be altered chemically [26]. Aside from carboxyl and hydroxyl groups, HA contains other reactive functional groups, including primary and secondary amines, which can be used to chemically conjugate with other molecules [27]. Furthermore, HA comes in various molecular weights and flexible chain lengths [28]. These structural features allow HA to be conjugated with polymers, peptides/proteins, lipids, and inorganic nanotemplates such as mesoporous silica, nanosheets, and carbon quantum dots for a variety of cancer therapeutic and diagnostic applications [[29], [30], [31], [32], [33], [34]].

HA conjugation has been widely explored for cancer drug delivery. HA conjugation with bovine serum albumin and liposomes is likened to a "trojan horse" strategy, enhancing the aggressiveness of loaded drugs through improved tumor tissue penetration and antitumor efficacy [35]. Conjugated HA nanoparticles have been employed for the co-delivery of multiple synthetic drugs [36,37], synthetic and natural drugs [[38], [39], [40]], and multiple natural drugs [41]. When complex with PGA, PEG, vitamin E, cholesterol, and others, HA-based nanoparticles can effectively deliver plant extracts while maintaining a nano size [[42], [43], [44]]. Conjugation of HA nanoparticles has shown promise in achieving multidrug resistance reversion [20,21,45], reduction of radioresistance [46,47], and prevention of postoperative tumor recurrence [48,49]. Furthermore, HA nanoparticle conjugation with mesoporous organosilicon is reported to "reeducate" tumor-associated macrophages (TAMs), making them more active against cancer cells [50]. Double targeting to CD44 receptor and oncofetal chondroitin sulfate [33] or CD44 and mitochondria [51] can also be achieved through conjugation with proteins/peptides.

Several review articles have discussed the use of HA nanoparticles in cancer therapy [[52], [53], [54], [55]]; nevertheless, they have not fully encompassed complete insights into the emerging trends and research gap in this field. Review styles are sometimes laser-like, typically analyzing a small pool of literature and providing a narrow picture. To address these constraints, bibliometric analysis emerges as an effective approach capable of incorporating a large number of articles, thus facilitating a more in-depth assessment, especially with machine learning [56,57]. Traditional bibliometric approaches rely primarily on keywords provided by authors or indexing agencies, which are often limited to 3–10 keywords. Notably, numerous important study variables are included in the abstract but not in the article's keywords, inaccessible by traditional tools. Incorporating machine learning techniques, notably natural language processing (NLP), represents an innovative path for improvement. Text mining enables the extraction of additional keywords and variables. NLP approaches designed specifically for scientific datasets like SciSpaCy have gained popularity. This program excels in extracting words or phrases (in a token form), expanding a dataset, and allowing for a more sophisticated exploration of key subjects [58,59]. Algorithms such as Latent Dirichlet Allocation (LDA), a generative probabilistic model of a corpus, help identify predominant topics [60,61]. Using these advances, we can better grasp current trends and research gaps in the field of conjugated HA-based nanoparticles. We additionally present a juxtaposition between the outcomes of traditional bibliometric analysis and NLP analysis.

## 2 Methodology

### 2.1 Search strategies and data collection

In our data search, we conducted a systematic search using specific keywords related to the complexation of HA-based nanoparticles with other materials for cancer therapy. The dataset was obtained from Scopus, a reputable academic database, and the information was downloaded in CSV format. To ensure that the topic within the dataset includes 'hyaluronic acid', 'nanoparticle', 'cancer', 'combination' and their synonyms. The keywords were set as *(TITLE-ABS-KEY ("Hyaluronic Acid" AND "Nanoparticle") AND TITLE-ABS-KEY ("Complex" OR "Combination" OR "Grafted" OR "Conjugated" OR "Modified") AND TITLE-ABS-KEY ("Cancer"))*. The initial search yielded 1242 documents, which were subsequently refined through a series of exclusion criteria.

The first criterion involved limiting the publication years to the range of 2014–2024, resulting in 1160 documents. This decade was chosen to focus on the most recent and relevant literature trends in the field. The dataset was narrowed to research articles, excluding 188 reviews, 6 book chapters, 8 conference papers, 2 surveys, and 2 editorial letters. Additionally, only documents in their final publication stage were considered, excluding 10 documents in the "articles in press" category because revisions may still apply to them. Literature selection also prioritized journal publications, excluding a ‘research article’ labeled document published by a book series. Furthermore, language criteria were applied to maintain language uniformity in the dataset for natural language processing, with a preference for English-language publications, excluding 10 Chinese language documents. This filter yielded 931 documents.

**Fig. 2:**
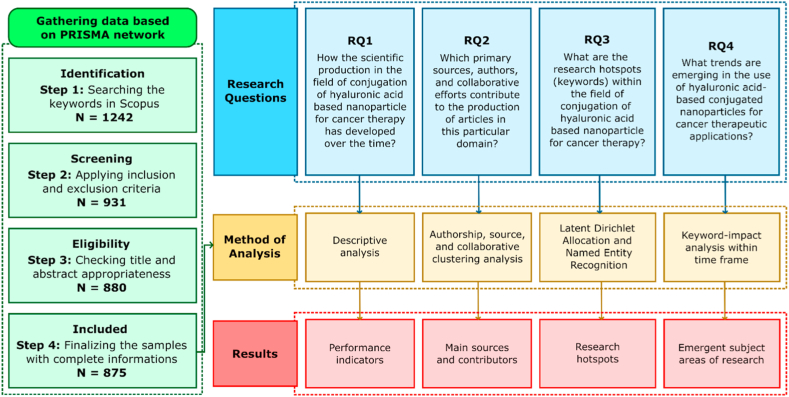
Flowchart of the methodology representing the searching strategies, yields, and research questions. RQ stands for research question.

To verify topic relevancy of the collected literature, the titles and abstracts of the publications were analyzed. Articles that are not about cancer therapy, do not involve the conjugation of HA, or do not result in nano-sized forms are subsequently excluded. Review articles passing the Scopus' web screening were eliminated, and materials lacking required metadata, such as abstracts and author/index keywords, were removed. The final dataset, which included 875 research articles, was a focused and relevant collection of literature on the subject. Fig. 2 depicts the PRISMA-based flowchart of search tactics and outcomes.

### 2.2 Text-mining, natural language processing, and data processing

In the bibliometric analysis, outcomes rely on keywords provided by authors or indexed keywords. These keywords typically do not encompass a broad range of study variables in the field, such as dosage form, drugs, conjugates, crosslinkers, surfactants, and other specific parameters crucial for supporting trend analysis. To identify keywords with these specific characteristics, text-mining was conducted in two ways: 1) collecting related keywords/categories through reading titles and abstracts, and 2) utilizing the Named Entity Recognition (NER) algorithm from SciSpaCy package on the final dataset. The NER method was executed through a custom Python module where filtering was applied to reduce non-noun words like 'efficient', 'increase', etc., and keywords used in search and dataset collection.

Newly mined specific keywords were stored in separate files based on categories, namely conjugates, API, particle shape, diagnostic methods, and type of therapy. These keywords were analyzed using the LDA method to discover the main topics. To make emerging topics more specific, keywords related to the inclusion criteria in the literature collection phase were excluded. A total of 20 topics were generated, and each had 5 keywords with a coherence score of 0.317.

In this study, certain processes that could not be executed using available tools were handled through custom Python modules. These processes involved translating a scanned and filtered database into a CSV format recognized by Biblioshiny. It also encompassed determining the frequency of occurrence of NER-mined keywords in the dataset, establishing the keyword timeframe dataset, and calculating the keyword impact using Equation (1).

1 $$Keyword\;impact=\frac{Citation}{Frequency}$$

The bar plot related to the topics generated by LDA was visualized using the Python packages pyLDAvis and matplotlib. Meanwhile, the timeframe, keyword impact, and radial network were visualized using the ggplot and Network3D package in RStudio (Version 4.2.1, RStudio Inc, Boston, USA).

### 2.3 Statistical analysis

Correlation analysis was conducted on several variables, such as the number of articles, H-index, G-index, and M-index, using Pearson's correlation method in RStudio.

## 3 Results and discussion

### 3.1 Overview

**Fig. 3:**
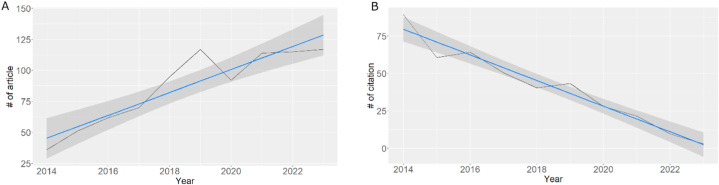
Dataset overview: (A) distribution of articles and (B) trend in article citations over time.

The collected dataset consists of research articles from 2014 to 2024, totaling 875 articles. These articles are sourced from 210 journals and involve 3797 authors. As depicted in Fig. 3A, the trend in the number of articles in the field of conjugating HA-based nanoparticles for cancer therapy and diagnosis shows a consistent upward trajectory over time. This indicates the sustained interest of researchers and continuous innovation within this field. A higher volume of research publications enhances the potential for discovering new insights and gaining a deeper comprehension of the subject matter. Moreover, the increasing number of publications suggests robust collaboration among researchers, with an average of 7.5 authors per article. Collaboration facilitates the convergence of diverse expertise and methodologies, contributing to a more diverse and innovative range of publications [62]. Moreover, the growth involves contributions from international researchers (16.57 %), which may signify that research in the field is becoming increasingly relevant and globally recognized, demonstrating an enhanced level of international engagement. Publications on the related topic appear to decline in 2019–2020 and rise again afterward. This could be attributed to the emergence of new topics that captured our attention during those years, particularly the COVID-19 pandemic.

The articles in this field receive an average of 33.39 citations, totaling 38,134 references. In Fig. 3B, it can be observed that the citation count decreases as we approach the present time. This is considered normal as articles become more recent. Typically, it takes around 2–10 years for articles to start accumulating citations [63]. The decline in citations may also indicate the maturity of the research field, where fundamental concepts and primary discoveries are widely accepted in the scientific community. This suggests a shift towards stability, with the research focus on novel areas or deeper intricacies [64,65]. Furthermore, the assessment of publication quality extends beyond mere citation numbers. Despite a reduced citation count, increasing average citations per article or journal impact factor indicates higher overall publication quality. On the other hand, the average age of the documents in our dataset was 4.6 years, categorizing them as relatively new/fresh to get a citation, as mentioned before.

### 3.2 Sources analysis

**Fig. 4:**
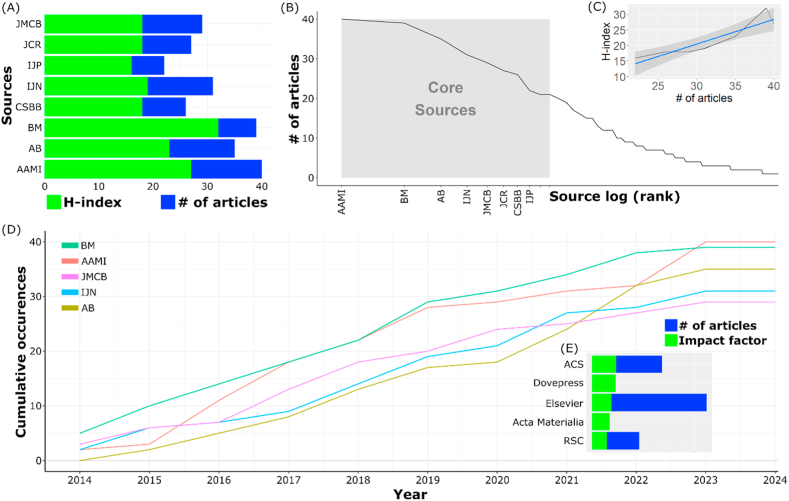
Sources landscape: (A) Distribution of scientific journals and their impact, (B) relevancy, (C) number of articles and H-index correlation, (D) source dynamics, and (E) publisher impact. Note: JMCB = Journal of Material Chemistry B, JCR = Journal of Controlled Release, IJP = International Journal of Pharmaceutics, IJN = International Journal of Nanomedicine, CSBB = Colloids and Surface B: Biointerface, BM = Biomaterials, AB= Acta Biomedica, and AAMI = ACS Applied Materials and Interfaces.

Results of the descriptive analysis indicate that 10 journals serve as the primary sources of articles on the discussed topic. So far, Biomaterials (BM) and ACS Applied Materials and Interfaces (AAMI) have emerged as key contributors to publications related to the topic based on the number of published articles, H-index, and Bradford's law (see Fig. 4A and B). An increase in the number of published articles correlates significantly with improved quality of publications, as evidenced by a rise in the journal's H-index (cor. value = 0.915, *p* < 0.01, see Figure XC). As seen in Fig. 3D, AAMI and BM also consistently increased the number of articles published on the related topic over time. A positive correlation between the number of articles and the H-index may suggest that the journal follows a focused and consistent approach in publishing research within the specific topic. This commitment disseminates impactful research in the specific field, contributing to the establishment of its academic standing and recognition within the scholarly community [66].

Based on the number of published articles, Elsevier Ltd. stands out as the publisher with the highest volume of publications on this topic. This is attributed to Elsevier's extensive array of journals covering diverse scopes, ranging from applied fields like pharmaceuticals to pure chemistry. However, when considering impact factors, ACS emerges as the publisher with the highest influence. Unlike Elsevier, ACS concentrates on publishing articles within the chemistry domain, fostering a strong community base that facilitates quicker absorption of its output on this topic. Following ACS, Dovepress also boasts a higher impact factor than Elsevier. Like ACS, Dovepress is a publisher focused on the medical field, contributing to its loyal user base. This demonstrates that consistency and a narrow scope at the journal and publisher levels lead to greater impact and easier assimilation by the community.

### 3.3 Authorship and collaboration network

**Fig. 5:**
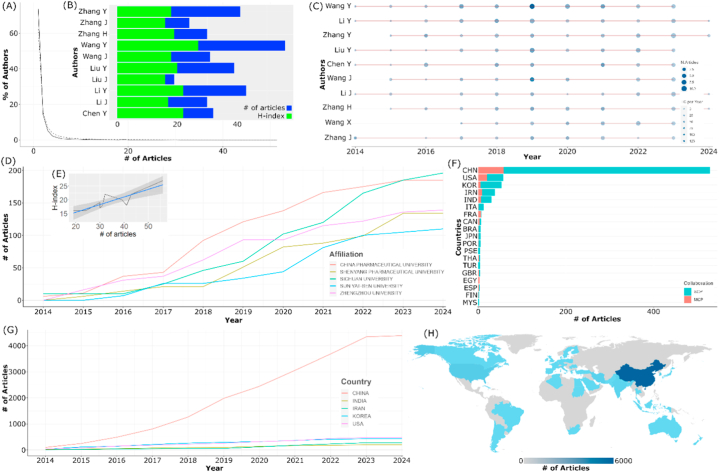
Authorship landscape: (A) Author productivity through Lotka's law, (B) author published articles and H-index, (C) author production and citation over time, (D) dynamics of affiliation production, (E) correlation of author production and H-index, (F) countries production, (G) dynamics of country production, (H) map analysis of country production. Note: SCP = single country publication, and MCP = multiple countries publication. The country code used in the figure is based on ISO3C.

Out of 3797 authors, only 0.2 % have published more than 20 articles on this topic (see Fig. 5A). The top 10 authors with the highest article count are depicted in Fig. 5B. Four of these ten authors continue to actively research and publish articles on this topic through 2024 (see Fig. 5C). Wang Y. stands out as the author with the highest number of articles. Furthermore, Wang Y. consistently publishes every year and has achieved the highest H-index on this topic. Similar patterns are observed in other authors, where the number of published articles correlates with their H-index. (cor. value = 0.859, *p* < 0.01, see Fig. 5E).

Viewing with a perspective of affiliations, contributions from China emerge as the most active communities researching this topic (see Fig. 5D, F, and 5H). The top 5 contributing affiliations all come from China, with the highest contribution coming from China Pharmaceutical University. This is not a coincidence; China consistently stands out as a major contributor across various fields [67]. In terms of quantity, China is not only active domestically (authorship originating solely from China) but also leads in publishing collaborative articles with other countries (see Fig. 5F), with a continuous increase in the number of publications per year on this topic (see Fig. 5G).

**Fig. 6:**
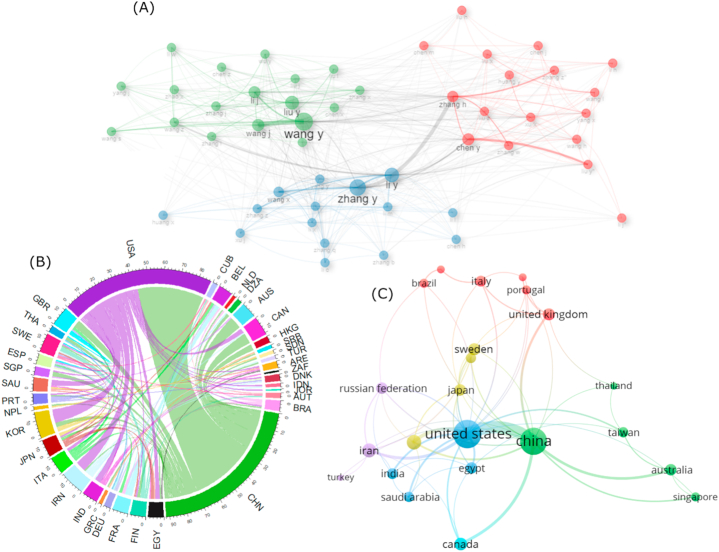
Network analysis of (A) co-authors' collaboration and countries' collaboration in chord diagram (B) and network diagram (C). These images were created by VOSviewer and RStudio. Note: The country code used in the figure is based on ISO3C.

Collaboration is dominated by three clusters, Wang Y, Zhang H, and Zhang Y. Each cluster consists of more than 15 members. These clusters exhibit collaborative relationships within and between clusters, sometimes involving direct collaboration among cluster members (excluding the center clusters), as depicted in Fig. 6A. China once again leads in international collaboration at the national level, with more than 90 articles, followed by USA with over 80 articles (see Fig. 6B). Collaboration patterns among countries form five clusters. The first cluster comprises China, Thailand, Taiwan, Australia, and Singapore, reflecting the collaboration of Asia-Pacific countries. The second cluster represents countries in North America and Middle East, also with India representing South Asia. The third cluster, Turkey, Iran, and Russia, indicates collaboration in the Eurasian region, connecting the Middle East, Central Asia, and Eastern Europe. Other clusters demonstrate cross-continental collaboration (see Fig. 6C).

### 3.4 Keyword analysis and trending topics

In this study, we used NER and LDA to identify crucial keywords based on the observed categories and parameters and uncover patterns between them to detect emerging subtopics. The NER method, powered by SciSpacy, made extracting scientific terms or phrases from abstracts and full articles easier, resulting in a dataset enriched with highly specific, topic-relevant keywords [58]. Meanwhile, LDA is a statistical model employed in bibliometric analysis to uncover hidden topics within large document collections [68]. Developed by Blei, Ng, and Jordan in 2003, LDA assumes that each document is a mixture of different topics, and each word in a document is generated from one of these latent topics. LDA facilitates automated topic extraction, clustering of documents, similarity analysis, and dimensionality reduction, making it particularly valuable for researchers in bibliometrics [60]. By applying LDA with specific and crucial keywords extracted using NER, scholars can accurately identify and analyze major subtopics in academic literature, track research trends, and understand the relationships between various topics across a substantial corpus of documents [69].

#### 3.4.1 Text-mined keywords by Named Entity Recognition

**Fig. 7:**
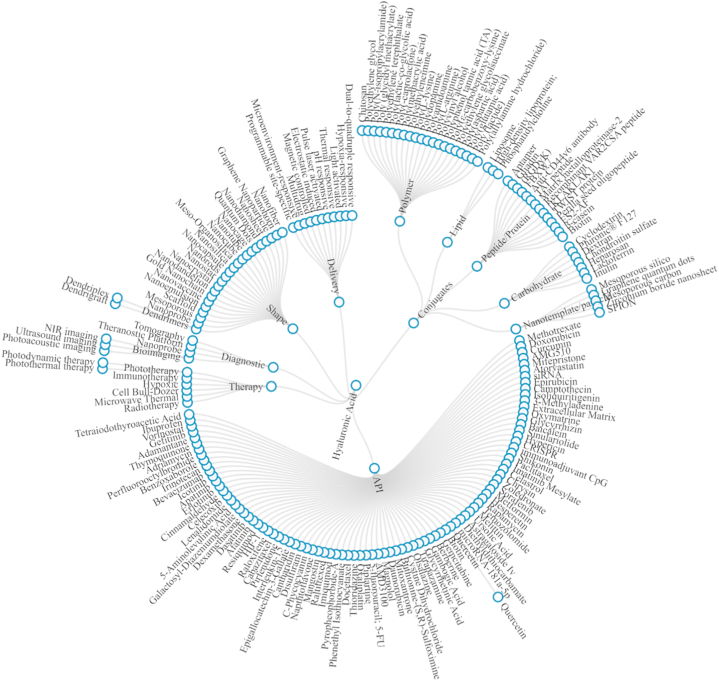
Characteristics of hyaluronic acid (HA)-based nanoparticles; Insights from manual text-mining on API, conjugates, therapy methods, diagnostic platforms, shapes, and delivery behaviors. This image was created by Network3D in RStudio.

After evaluating the titles and abstracts, numerous key pieces of information about the properties of HA-based nanoparticles were discovered that would not have been revealed by a general bibliometric analysis. These elements include active pharmaceutical ingredients (API), conjugates, therapy approaches, diagnostic platforms, morphologies, and delivery behaviors (Fig. 7). Because the primary focus of this article is the alteration of HA via conjugation, a more in-depth examination of many forms of conjugates has been carried out. Polymer, lipid, peptide/protein, carbohydrate, and nanotemplate are the most prevalent types of conjugates utilized in conjunction with HA.

##### 3.4.1.1 Anticancer agents

Of the 875 articles in the dataset, 263 (30 %) utilize DOX. Following this are PTX (n = 67), siRNA (n = 65), epirubicin (n = 39), curcumin (CUR) (n = 35), and camptothecin (n = 30). DOX is frequently utilized in cancer research for several reasons. Its proven effectiveness against various cancer types, including breast cancer [[70], [71], [72], [73]], leukemia [74], brain [75,76], thyroid [77], prostate [78,79], and lung cancer [80,81], makes it a popular choice for seeking insights into potential efficacy across diverse malignancies. It intercalates into DNA, inhibiting topoisomerase II, which disrupts DNA replication and transcription, leading to apoptosis [3]. It also generates reactive oxygen species (ROS), causing oxidative damage to cellular components, including DNA, proteins, and lipids. This oxidative stress contributes to both cancer cell death and the drug's cardiotoxicity [82]. Additionally, DOX induces mitochondrial dysfunction, releasing cytochrome C, which activates caspases and triggers intrinsic apoptosis [83]. It further inhibits NF-κB, reducing anti-apoptotic signaling, and blocks telomerase activity, promoting telomere shortening and cell senescence [84]. These combined mechanisms make doxorubicin a potent chemotherapeutic agent. On the other hand, Paclitaxel stabilizes microtubules and prevents their depolymerization during cell division, halting mitosis and leading to cancer cell death [85]. It has been a critical drug for treating solid tumors, including breast, ovarian, and lung cancers, since the 1990s [86]. PTX's unique mechanism and its effectiveness in combination therapies make it a standard in cancer research. However, challenges posed by DOX and PTX toxicity, particularly to normal tissues and systemic toxicity, prompt research efforts to mitigate these side effects or develop clinically more acceptable formulations [[87], [88], [89]]. Studies often focus on combinations with other chemotherapy agents or therapies to enhance therapeutic effects and overcome drug resistance [90,91].

Small interfering RNA (siRNA) is extensively explored for its potential role in cancer therapy due to its precision in selectively silencing specific genes involved in cancer development [20,92,93]. This molecular tool offers a targeted approach, minimizing off-target effects and reducing harm to healthy cells [94,95]. The ability to design siRNAs tailored to individual genetic characteristics holds promise for personalized cancer treatment [96]. Moreover, siRNA addresses challenges such as drug resistance by targeting key genes associated with resistance mechanisms [[97], [98], [99]]. Its compatibility with combination therapies, such as chemotherapy or immunotherapy, enhances treatment efficacy, making it a versatile candidate in cancer research [98,100,101]. Additionally, compared to conventional chemotherapy, the reduced systemic toxicity of siRNA makes it a preferred option [20,102]. The effectiveness of several siRNAs has also entered phase 1 and 2 clinical trials. These include ALN-VSP02 and DCR-MYC for solid tumors [103,104], Atu027 and siG12D LODER for pancreatic cancer [105,106], TKM- PLK1 for adrenal cortical carcinoma [107], and NBF-006 for non-small cell lung cancer [108]. siRNA is water-soluble and easy to formulate, but its main challenges are stability and bioavailability. Its gene-silencing role often leads to its combination with chemotherapy drugs to prevent resistance. It is commonly formulated into dual-compartment nanoparticles, like liposomes and co-polymer systems with surface modifier, discussed in the next section to address these challenges.

CUR, derived from turmeric, and camptothecin, particularly in its derivatives like topotecan and irinotecan, stands out as our dataset's first and second most researched natural compounds. CUR's appeal lies in its potent anti-inflammatory and antioxidant properties, addressing key factors in cancer development [[109], [110], [111]]. Its ability to modulate multiple signaling pathways involved in cell growth and survival makes it a multifaceted candidate for cancer treatment [112]. On the other hand, camptothecin's notable mechanism involves inhibiting topoisomerase I, which is crucial for DNA replication, effectively preventing cancer cell proliferation [113,114]. Its potent antitumor activity across various cancer types has fueled extensive research efforts. However, CUR and camptothecin share similar pharmacokinetic challenges that limit their clinical use. Both compounds suffer from poor aqueous solubility, leading to low bioavailability, especially when administered orally [115,116]. They also undergo rapid metabolism and clearance, primarily through glucuronidation and sulfation in the liver, resulting in a short half-life and diminished therapeutic effectiveness [117,118]. Both are chemically unstable: curcumin degrades quickly at neutral-basic pH [119], while camptothecin's lactone ring hydrolyzes into an inactive form [120].

##### 3.4.1.2 Conjugates, nanoparticle shapes, delivery mechanisms, and types of therapies

The most frequently utilized polymer as a conjugate is polyethylene glycol (PEG) (n = 78), followed by chitosan (n = 67), poly(lactic-co-glycolic acid) (n = 45), polydopamine (n = 32), polyethyleneimine (n = 31), polyamidoamine (n = 14), and poly(caprolactone) (n = 9). PEG is often chosen due to its ability to enhance the circulation of nanoparticles in the blood through "PEGylation," reducing opsonization by an immune system and extending a half-life of nanoparticles [121]. Some hydrophobic drugs, including those containing metals, are also PEGylated to improve their solubility [122]. PEG is sometimes employed in multidrug delivery as a conjugate because it readily interacts with biomolecules like an extracellular matrix. In addition to improving drug circulation and bioavailability in tumor tissues, PEG prevents systemic toxicity [123]. PEG inhibits immune recognition and has been specifically reported to target melanoma [124]. Conjugation of PEG to fasudil compounds to form micelles directly allows for controlled release with UV light assistance [121]. PEG is also utilized in ChitoPEG/DOX/IO nanocomposites to produce nanoparticles responsive to GSH and magnetic stimuli [19]. Transfection is also reported to improve with PEG conjugation [125]. Its compatibility with various compounds makes PEG the most commonly used conjugate in HA-based nanoparticles.

Chitosan is a thermoresponsive and pH-responsive component, allowing faster drug release at higher temperatures common in tumor tissues [126,127]. Chitosan-coated nanoparticles effectively accumulate in tumor tissues, and the nanoplatform is activated by a weakly acidic tumor microenvironment, leading to a release of ATV [128]. Chitosan is also enzymatically degraded and reported to control the drug release in a sustained manner [129]. Chitosan also serves as a compartmental separation layer in a multidrug delivery system [81,93,130]. Chitosan contributes to the bioadhesive potential of nanoemulsions and its ability to maintain breast tissue retention for a longer duration without causing undesirable histological changes [131]. HA nanoparticles conjugated with chitosan exhibit excellent stability in blood circulation and can deliver drugs into breast cancer cells with a responsive nature to intracellular environments, such as a presence of glutathione (GSH), hyaluronidase (Hyals), and acidity (pH 5.0) [[132], [133], [134]]. Chitosan can also be utilized to create a layer-by-layer nanoarchitecture system to control a slow release of drugs through erosion of each layer [135,136].

Poly(lactic-co-glycolic acid) (PLGA) serves as a biocompatible and biodegradable polymer used in the fabrication of nanoparticles, contributing to controlled drug release and targeted delivery in cancer therapy [137,138]. It also contributes to the stability of the nanoparticle dispersion [16,139]. PLGA allows a zero-order degradation-controlled release on self-assembled HA–PLGA nanoparticles [140]. PLGA is also employed in the fabrication of nanoparticles modified with a platelet membrane to deliver bufalin to liver cancer cells. These nanoparticles are designed to enhance tumor accumulation through enhanced permeability and retention effect (EPR) and actively target cancer cells through biomimetic platelet membranes [141]. Using recombinant human hyaluronidase PH20 with these complexed nanoparticles improves nanocarrier diffusion in solid tumors [142].

Polydopamine is often transformed into a mesoporous structure for loading metal-based materials [111,143,144] or to support photothermal and photodynamic therapy [[145], [146], [147], [148], [149], [150]]. Photothermal therapy (PTT) uses photothermal agents to convert near-infrared light into heat for localized cancer cell destruction [151]. In contrast, photodynamic therapy (PDT) employs light-activated photosensitizers to produce reactive oxygen species that cause oxidative damage and cell death in cancer cells [152]. On the other hand, polyamidoamine dendrimer is utilized as a highly branched biphasic polymeric structure in synthesizing various biomolecules or metallic nanoparticles. They play roles as both reducing and stabilizing agents in a one-pot chemical method on a nanoplatform [[153], [154], [155], [156], [157], [158], [159]].

Liposome has been consistently employed as a vital component within an HA-based nanoparticle system. For instance, USP22 siRNA-loaded nanoliposome conjugated with CD44 antibody (USP22-NLs-CD44) was designed to effectively deliver USP22 siRNA to CD44^+^ gastric cancer stem cells [160]. The combination of liposomes with magnetic nanoparticles (MNP) in a bubble-generating magnetic liposome (BML) exhibited promise for magnetic field-directed targeted drug delivery in cancer therapy [161]. Liposome was also integrated into a nano-vehicle for dual-targeted (magnetic and ligand) and dual-mode (photothermal and photodynamic) cancer therapy, demonstrating enhanced cytotoxicity to human glioblastoma cells [162]. Another innovative approach involved the development of a novel nano-complex combining thioglycolic acid-conjugated chitosan nanoparticles, biomimetic transfersome, and amphiphilic HA for effective VEGF siRNA delivery [163]. Liposome coated with a multivalent immunoadjuvant (HA-CpG) and loaded with a fluorophore (IR-7-lipo) was demonstrated as an endogenous vaccine platform for combinatorial photothermal ablation and immunotherapy [164]. An HA-modified transfersome was also prepared for transdermal drug delivery, indicating enhanced lymphatics absorption and improved tumor cell uptake [165,166]. Finally, PEG-HA-coated liposomal complexes were demonstrated as a promising siRNA delivery system, effectively adjusting solid tumor P-glycoprotein expression and potentially reversing multidrug resistance in breast cancer therapy [45,101].

The utilization of peptides and proteins in conjugation with HA-based nanoparticles has been a focal point in developing innovative cancer therapies. For instance, research by Xiang C (2021) explores the conjugation of HA and KLA peptide, targeting receptors for HA-mediated motility isoform B (RHAMMB). RHAMMB, a protein considered undetected in most adult tissues, is highlighted in developing nanoparticles targeted to pancreatic neuroendocrine tumors (PNETs) [167]. A similar approach can be found in the study by Ruizhi X. et al. (2019) [51], where dual-targeted nanoparticles (HKHD) are composed using HA as a modification on hollow carbon nitride nanospheres (HCNS). HKHD efficiently delivers DOX to mitochondria and tumor cell nucleus using mitochondria-localizing peptide (KLA) and HA. Integrating the mitochondria-targeting peptide in these nanoparticles enhances effective photodynamic and photothermal effects to inhibit tumor growth.

A similar context is also evident in the drug delivery system developed by Chen S. et al. (2017) [168], where the cationic peptide R8 and pro-apoptotic peptide TPP-KLA are decorated on gold nanostar nanoparticles (AuNS) through Au-S binding. Peptides R8 and TPP-KLA balance drug delivery to tumors through CD44 receptor recognition and induce a photothermal effect at a mitochondrial level for combined photothermal therapy and chemotherapy. These examples indicate that conjugating peptides and proteins with HA nanoparticles is an intriguing therapeutic strategy, enabling effective drug delivery to tumors with higher specificity.

Conjugation with β-cyclodextrin (β-CD) has been crucial in HA-based nanoparticle therapy. β-cyclodextrin constructs supramolecular nanoparticles that are light-responsive and targetable, involving host-guest interactions between β-CD and HA [169]. The presence of β-CD not only enhances the biocompatibility of these supramolecular nanoparticles but also provides the ability to specifically load and release hydrophobic anticancer drugs, such as camptothecin, due to the light-responsive capability of the 2-nitrobenzyl ester group. Supramolecular HA-β-CD complex containing adamantane exhibits higher cytotoxic activity against CD44-positive cancer cells [170]. β-CD imparts colloidal stability and chargeability to nanoparticles and reinforces their cancer cell-targeting properties through conjugation with HA.

The significance of β-CD in cancer therapy is evident in the research by Yuanyuan Z. et al. (2018) [171], where supramolecular nanoparticles composed of β-CD conjugated with HA and camptothecin prodrug show great potential for combination photodynamic therapy and chemotherapy. The use of β-CD conjugated with HA provides the ability to generate reactive oxygen species (ROS) in response to light exposure. At the same time, conjugation with HA enhances its targetability through CD44-mediated endocytosis. Xin C. et al. (2016) and Shazid MS. et al. (2015) also reported that supramolecular nanoparticles consisting of HA and α/β-CD formed a drug delivery matrix responsive to pH, thermal stimuli, and external control [172,173]. This system combines the influence of acidity for pH-regulated drug release with the photothermal capability to stimulate excess release of anticancer drugs. Integrating internal pH/thermal responsiveness and external NIR control makes this system a promising approach for tailored chemotherapy release based on physiological needs.

**Fig. 8:**
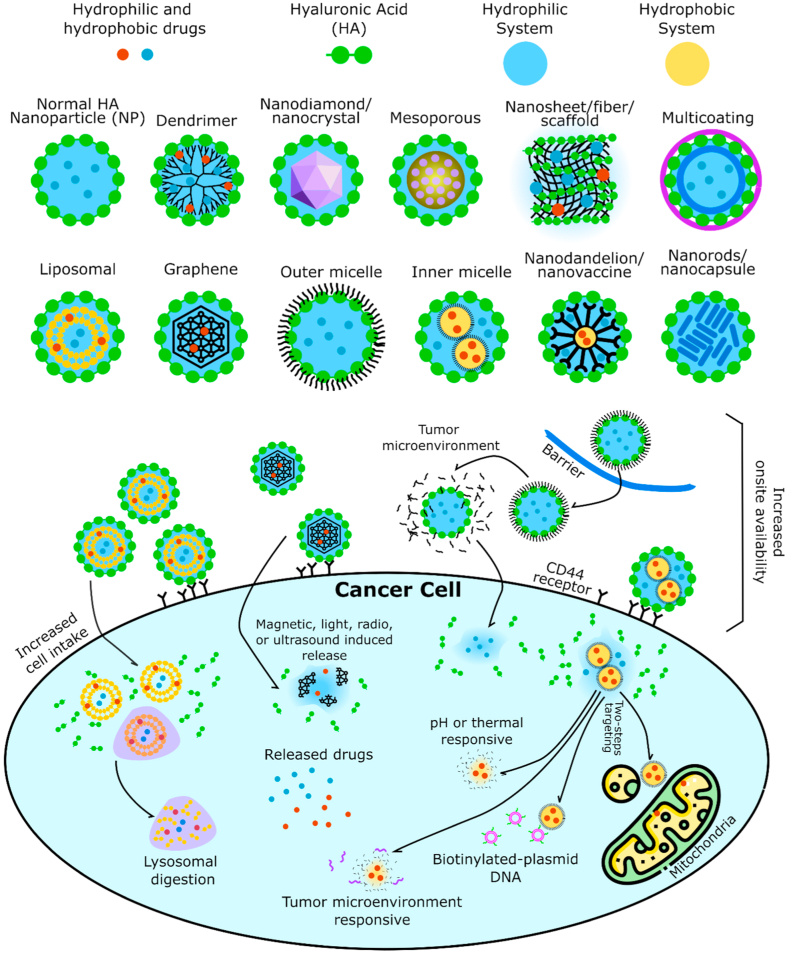
Conjugated hyaluronic acid (HA) based nanoparticle delivery systems and their release mechanism.

The unique properties of mesoporous silica nanoparticles (MSNs), particularly their high surface area and well-defined mesoporous structure, make them an ideal platform for drug loading and release [129,[174], [175], [176]]. Incorporation of MSNs into the nanoplatforms allows for stimuli-responsive drug release, such as pH, enzyme, and redox-triggered mechanisms, providing an efficient means for drug delivery into a cytosol of cancer cells [23,77,[176], [177], [178]]. Moreover, the multifunctionality of MSNs is demonstrated in several studies where they are employed for drug delivery and imaging agents loader, enhancing theranostic capabilities of the nanoparticles [174,175,179,180]. Convenient modification of MSNs provides opportunities for constructing a new generation of nanocarriers with multiple functions, exemplified by a multifunctional drug delivery system based on MSN capped by a gadolinium-based bovine serum albumin complex and HA [181]. Other polymers are utilized as crosslinkers with drugs, metals, or RNA due to their properties, such as stable dispersion, biocompatibility/hemocompatibility, pH sensitivity, and good adhesiveness [137,[182], [183], [184]]. Refer to Fig. 8 for an illustration of nanoparticle morphology and their release mechanisms.

#### 3.4.2 Latent Dirichlet Allocation

**Fig. 9:**
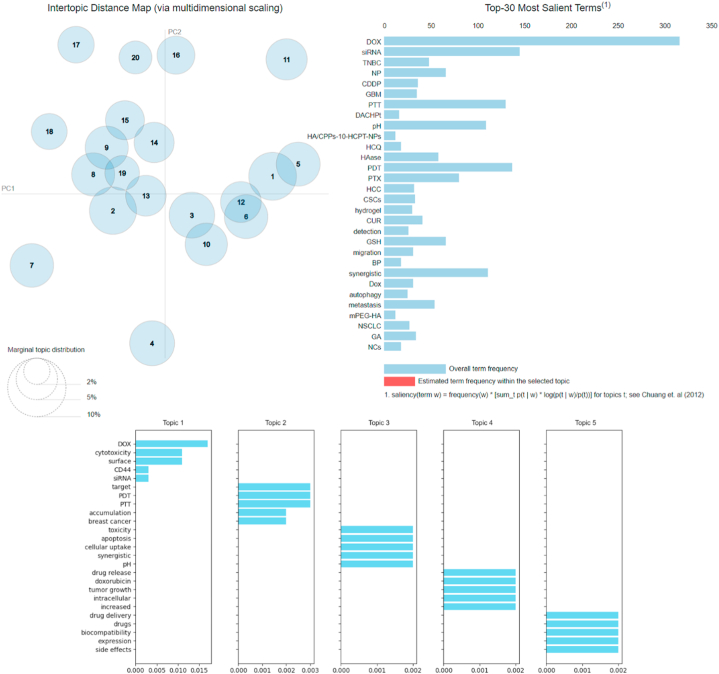
Top 30 most salient keywords and top 5 topics in the field. These images were created by pyLDAvis in Python.

Based on the dataset screening results, the top 30 terms that emerged are keywords that the NER algorithm identified as having the highest frequency. For example, DOX was mentioned over 300 times in the texts. This figure approximates the NER estimate, albeit slightly higher. This difference could be due to the double counting of multiple mentions inside a single article. LDA approach is likewise case-sensitive [185], discriminating between “DOX” and “dox”, as illustrated in Fig. 9. Furthermore, the results are comparable to NER, identifying the most often utilized active anticancer drugs in the order DOX > siRNA > PTX > CUR. Triple-negative breast cancer or breast cancer is the primary disease target in the dataset and is the third top term. The same applies to other top keywords. These results indeed indicate that LDA method can bring forth topics based on combinations of keywords that frequently emerge during our manual screening. Out of the 20 generated topics, we selected the top 5 topics with unique keywords and high relevance scores or λ (lambda) value.

The first topic contains dox, cytotoxicity, surface, CD44, and siRNA keywords. It encapsulates the context of applying surface modification to target the CD44 receptor to deliver DOX, siRNA, or their combination to enhance cytotoxic effects. We constructed it in this way as we excluded non-noun words in the dataset, such as effective, efficient, etc., and applied inclusion criteria and search keywords like HA, conjugation, nanoparticle, etc., since these keywords already clearly describe the core topic of the entire dataset and aim to enhance the appearance of other objects/subjects as trend varieties within that core topic. This topic is crucial as it refers to the application of HA as a ligand targeting CD44 to maximize the cytotoxicity of anticancer agents, with DOX and siRNA being the most frequently used (based on NER and LDA studies).

The second topic is more interesting and specific. It includes keywords such as target, photodynamic therapy (PDT), photothermal therapy (PTT), accumulation, and breast cancer. This topic illustrates using PDT and PTT methods to target the high accumulation of anticancer agents in breast cancer cells. PTT and PDT themselves are the most frequently used therapy methods, with usage frequencies of 168 and 123, respectively, followed by immunotherapy (n = 100), hypoxic conditioning (n = 44), and radiotherapy (n = 18).

The third topic contains keywords such as toxicity, apoptosis, cell uptake, synergistic, and pH. They can be assembled into topics related to mechanisms or strategies to enhance apoptosis and toxicity effects. Synergistic combination therapies, controlled release regulation through pH responsiveness, and increased cell uptake can enhance therapeutic efficiency, leading to better on-site toxicity and apoptosis. This topic also has some connection to the fourth topic, including drug release, DOX, tumor growth, intracellular, and increased. This topic can be developed as follows: controlled release of DOX can enhance its intracellular availability, suppressing tumor growth. The fifth topic discusses drug delivery, drugs, biocompatibility, expression, and side effects. If associated, this could be interpreted as anticancer drug formulations using delivery systems that can improve biocompatibility and reduce side effects. This is a primary challenge in chemotherapy development, where systemic or local toxicity can affect healthy cells.

**Fig. 10:**
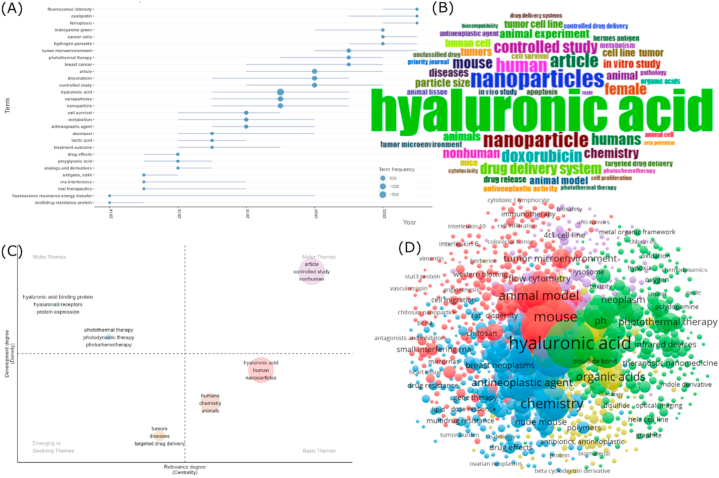
Conventional bibliometric analysis results. (A) Topic trends, (B) keyword thematic map, (C) keyword cloud, and (D) keyword clusters. These images were created by Biblioshiny.

In contrast to using machine learning algorithms, conventional bibliometric tools generate keywords that are more random and broadly meaningful, such as chemistry, animal model, mouse, organic acid, biosafety, protein, and others, making it difficult to assemble them to find representative main topics within the dataset (see Fig. 10A–D). NER + LDA is more effective at generating a set of interconnected keywords that are easier to link, helping reveal highly relevant topics from the dataset. Nevertheless, the use of such tools remains relevant, particularly for visualization in datasets previously filtered and reduced in dimensions. Biblioshiny, VOSviewer, and CiteSpace are excellent at producing visually appealing and colorful data visualizations. Additionally, the topic trends feature can still be used in analysis to observe the timeline of using specific keywords. As seen in Fig. 10A, although not among the top keywords, oxaliplatin is a drug that began to be used in the related topic (conjugated HA-based nanoparticle) since 2021. Oxaliplatin itself is an adjunctive anticancer drug used to manage and treat metastatic colorectal cancer stage III [186]. Similarly, ferroptosis (since 2020) and tumor microenvironment (since 2019) are emerging in this topic. Distinct from apoptosis, necrosis, and autophagy, ferroptosis is a form of intracellular iron-dependent cell death. Extensive research indicates its pivotal role in tumor suppression, presenting new prospects for cancer therapy [187]. The tumor microenvironment encompasses everything in the tumor environment, including cancer cells, stromal tissue, and cellular matrix [188]. While Rudolf Virchow has coined the term since 1863 [189], it may have been used recently to target and control drug release in this topic. For example, GSH targeting has been utilized since 2018 [163], when it is known that GSH is one of the tumor microenvironment components.

### 3.5 Impact-based trends in conjugated HA-based nanoparticles

In this trend analysis, the time variable is involved to observe the dynamics of subtopics within the main topic. The frequency of keyword appearances over time can reveal emerging subtopics. However, this method can also be biased by standardization or perception alignment. For example, frequently used broad-spectrum anticancer drugs in this topic are DOX and PTX, which are widely known as standard anticancer agents and commonly used in research as positive controls. It is not because they are better than other therapeutics. Still, they have been established as benchmarks, indicating that the development of new drugs should surpass existing standard drugs in the market. Consequently, their increased use in a field is not based on trends or researchers' interests but is merely used as a reference in studies. Hence, this trend analysis should involve parameters of interest or absorption capacity, one of which can be depicted by citation patterns on specific keywords, indicating an interest of other researchers in using them as a reference.

#### 3.5.1 Trends in active pharmaceutical ingredients (APIs)

**Fig. 11:**
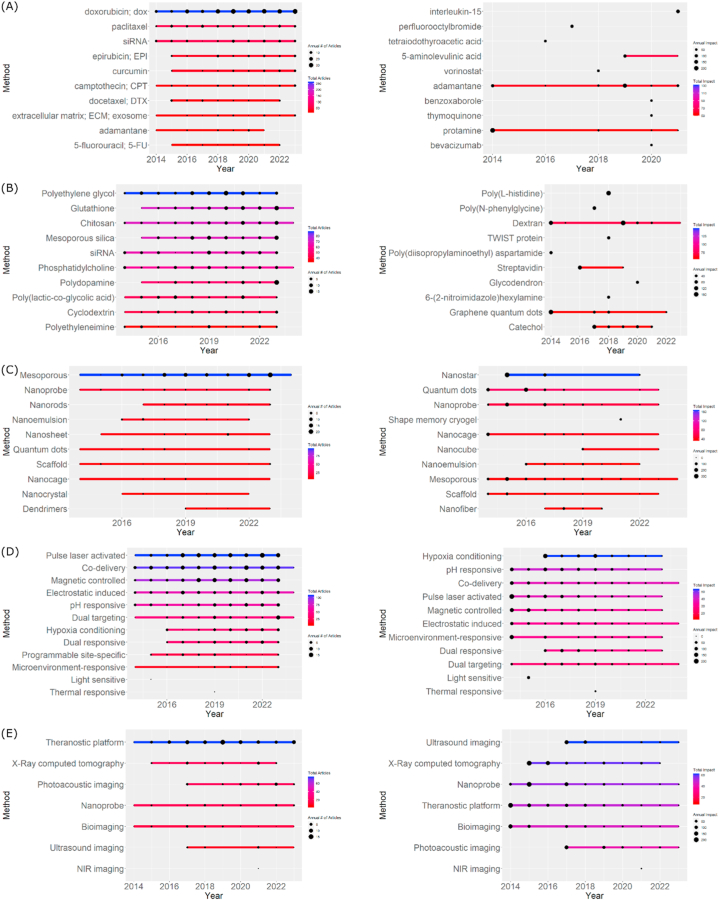
Keyword trends based on usage frequency (left) and keyword impact (right) of (A) active anticancers, (B) conjugates, (C) shapes, (D) method of therapies, and (E) method of diagnostic.

If we plot keyword impact vs. time, the trends can significantly differ (other APIs showed up as emerging drugs in this topic). For example, based on keyword impact, a trend emerges for drugs such as interleukin-15 (IL-15), perfluorooctylbromide, and tetraiodothyroacetic acid (tetrac). Their applications in HA-based nanoparticles are relatively new but have garnered substantial citations (Fig. 11A). IL-15 has been reported to have a potent therapeutic effect by stimulating cytotoxic T cells and activating natural killer (NK) cells [190]. This therapy has lower side effects compared to those of other agents. Perfluorooctyl bromide is developed for regulating cancer immunotherapy, functioning as a nano-agent for dual magnetic resonance imaging (MRI)/CT imaging and advanced immunogenic cell death (ICD) [191].

On the other hand, tetrac is a thyroid hormone modulator that may have anti-proliferative effects on certain cancer cells by inhibiting the activity of thyroid hormone receptor beta (THR-β), which regulates cell growth and proliferation [192]. Despite receiving numerous citations, these three APIs have not been widely adopted in the following years, so their trends cannot be considered. They have been adopted into clinical trials and applications in single form or other delivery systems. However, their use in HA-based conjugated nanoparticles has only been recently studied and still requires time before progressing to clinical trials. In contrast, 5-aminolevulinic acid (5-ALA), adamantane, and protamine have continued to be used since their initial application in these nanoparticles and have consistently received numerous citations.

Photodynamic therapy (PDT) is an innovative method in cancer treatment where 5-aminolevulinic acid (5-ALA) is used as a photosensitizer compound. The therapy process begins with administering 5-ALA into the target cells, which is then activated through laser light at a specific wavelength. This activation triggers chemical reactions, producing ROS and other reactive species [193]. These effects can damage cancer cells, providing a specific therapeutic approach as 5-ALA can concentrate more on cancer cells than normal cells. This advantage makes 5-ALA a preferred choice in PDT. The use of 5-ALA in PDT is not limited to therapeutic effects but also involves a role in diagnostic imaging. As a contrast agent, 5-ALA has been applied in an endoscopy examination for tumor detection and visualization, enabling early detection and more accurate targeting in cancer treatment [194]. Chiang CS et al. (2021) explored the role of 5-aminolevulinic acid (5-ALA) in targeted sensitization-enhanced radiotherapy (TSER), a strategy combining 5-ALA with the radio enhancer GoldenDisk (GD) for precise cell-targeted radiotherapy in glioblastoma multiforme (GBM). Incorporating 5-ALA enhances a radiosensitization effect, resulting in DNA damage and increased ROS levels, sensitizing GBM cells to radiation. This approach has a potential for low-dose precision radiotherapy, improving outcomes for GBM patients [195]. Yaping W et al. (2018) reported the role of 5-ALA in a chemo-photodynamic dual therapy for breast cancer. Conjugated onto polysaccharide-based nanocomplexes, 5-ALA contributes to enhanced cellular uptake and combined anticancer efficiency. The study showcases the potential of 5-ALA in targeted combination therapy [196]. Another study highlights using 5-ALA in a multifunctional theranostic nanoplatform (GNR-HA−ALA/Cy7.5-HER2) for breast cancer therapy, where 5-ALA is one of the PDT components. The nanoplatform was demonstrated for dual-targeted and fluorescence imaging-guided PDT/PTT against HER2-positive breast cancer with enhanced therapeutic efficacy [197].

In recent research, adamantane has been used to develop nanomaterials for cancer therapy and photodynamic applications. As part of a supramolecular assembly, adamantane-poly pyridyl ruthenium acts as a photosensitizer, playing a crucial role in the noninvasive regulation of excited-state oxygen generation [198]. Adamantane modification on hemin enhances the photocatalytic catalysis efficiency in Bi2Se3 nanoreactors, producing high oxygen reactivity for cancer therapy [199]. In the formation of supramolecular nanoparticles with a prodrug camptothecin and a photosensitizer porphyrin, adamantane participates in a structure responsive to reducing conditions by activating controlled drug release and generation of excited-state oxygen under light irradiation [200]. Overall, adamantane plays a central role in shaping supramolecular assemblies, enhancing stability, enabling controlled release, and contributing to the overall therapeutic efficacy in cancer therapy and photodynamic processes.

Protamine, a type of polypeptide found in salmon sperm and several other species, has garnered attention in anticancer research due to its potential properties. Studies indicate that protamine has an antiproliferative effect through its ability to interact with nucleic acids, inhibit DNA synthesis, and influence the growth of cancer cells [[201], [202], [203]]. Additionally, protamine's antimicrobial properties can provide protection against infections, which is critical for cancer patients with a declining immune system [204]. Protamine can also be transformed into a drug delivery vector [205], enhancing treatment effectiveness by delivering an active substance into cancer cells. The interaction of protamine with cell membranes and its potential as a radiation sensitization agent are focal points of research efforts aimed at improving the efficiency of anticancer therapy. Several studies have utilized protamine to deliver siRNA to inhibit multiple genes in various types of cancer [[206], [207], [208]]. Protamine is employed in delivering siRNA to suppress TGF-β levels in a tumor microenvironment [101]. It is also used to deliver CUR while simultaneously facilitating theranostic imaging with its radiation-sensitive properties [209].

Observed trends of these APIs indicate their current utilizations, which focus on harnessing structural flexibility for easy complexation with other API agents and nanoparticle systems. This imparts an adjuvant effect through direct inhibition of specific genes or pathways, either directly or indirectly, through gene silencing that interferes with the main agent's therapy. The radiosensitizing property is highly advantageous in combination therapies such as PDT/PTT/chemotherapy. Due to their biodegradable nature and sensitivity to pH, these APIs serve as a platform for dual targeting or dual responsiveness, aiding other therapeutic agents in reaching more specific areas. From this, it is evident that the current research direction of HA-based nanoparticle therapy for cancer no longer focuses on optimizing active substances' toxicity but on specific targeting strategies and precise release mechanisms to minimize systemic toxicity and impact on healthy tissues.

#### 3.5.2 Trends in conjugates

Dextran, streptavidin, catechol, and quantum dots are commonly used conjugates in the formulation of HA-based nanoparticles (see Fig. 11B). As a polysaccharide, Dextran offers high biocompatibility, reducing the risk of immune reactions or body rejection [210]. Its hydrophilic nature enhances the stability and solubility of nanoparticles in aqueous media, particularly for an intravenous route [211]. Its ability to easily form nanoparticles allows tuning of size and property to meet a need. The low cost and widespread availability of dextran also support the mass production of drug nanoparticles at an affordable cost [212].

Dextran was used as a base material for a biocompatible micellar nanoparticle (PCL-NPs), which integrates a chemiluminescence agent luminol, a photosensitizer chlorine e6 (Ce6), and a prodrug paclitaxel (PTX) to enhance the efficiency of combination therapy [213]. In another study, dextran was modified on hyaluronidase enzyme (HAase) to form a nanoparticle (DEX-HAase) that could trigger the breakdown of HA in the tumor extracellular matrix and enhance the therapeutic response of PDT [214]. Moreover, dextran was employed as a coating on superparamagnetic iron oxide nanoparticles (SPIONs) and in the formation of multilayer polyelectrolyte coatings on solid lipid nanoparticles (SLNs), with objectives to improve stability, molecular target properties, and drug circulation half-life [136,215].

Streptavidin is utilized in cancer therapy, and its application is in radionuclide therapy, where a radioactively labeled compound bound to biotin is conjugated with streptavidin [216]. Streptavidin exhibits a high affinity for biotin, enabling specific binding to cancer cells that have been modified to express biotin or after the administration of biotinylated compounds into the body [217]. Accumulation of radioactively labeled compounds in cancer cells through streptavidin-biotin interaction allows for the delivery of radioactive species to cancer cells, with the potential to damage DNA and produce therapeutic effects in cancer treatment. In the study by Nathan B. et al. (2019), streptavidin was used to bind plasmid DNA modified with biotin nucleotides, creating a specifically directed nanoparticle-plasmid complex. Streptavidin connects the plasmid and the nanoparticle, forming a structure that prevents premature dissociation between the nanoparticle and plasmid DNA [218]. Additionally, streptavidin conjugation accelerates drug release in the presence of Haase and biotin in vitro [219].

With its hydroxyl groups capable of interacting with heavy metals such as iron and copper, catechol becomes crucial in nanoparticle design for cancer therapy, focusing on heavy metal binding and specific targeting. Due to its ability to bind heavy metals, catechol has been widely used in forming gold nanoparticle delivery systems for combination chemo-photothermal therapy and pH/NIR-induced controlled release [220,221]. In the context of targeting, catechol can be utilized to modify a nanoparticle surface to specifically enhance binding affinity with a cell surface. Catechol is employed in a hydrogel nanocomposite to enhance structural stability and sustained local release of DOX in an oral cavity through excellent mucoadhesive properties of catechol [222]. Cheong AC et al. (2018) demonstrated that catechol in a fluorescent carbon nanoparticle delivers a redox and pH-responsive MnO_2_ nanosheet [223].

From the conjugate materials above, it can be observed that the direction of HA-based nanoparticle conjugation does not only cover CD44 receptor delivery but also includes combination therapy such as PDT/PTT/chemotherapy with site-specific dual targeting, dual responsiveness, or their combination. Moreover, these conjugates are utilized to stabilize the nanoparticle and enhance drug/metal loading through complexation.

#### 3.5.3 Trends in nanoparticle shape

Nanostars, particularly gold nanostars (AuNSs), are favored in cancer therapy due to their distinctive star-shaped morphology, which imparts several advantageous features (see Fig. 11C). The star-like structure provides an increased surface area, enhancing the loading capacity for therapeutic agents and promoting efficient drug delivery. Moreover, the multiple branches of nanostars facilitate strong light absorption, making them effective photothermal nanosystems when exposed to near-infrared (NIR) light [224]. This property is harnessed for PTT, enabling precise and controlled ablation of cancer cells. Additionally, the sharp tips of nanostars create localized electromagnetic hotspots, leading to enhanced photoacoustic and photoluminescence signals, aiding in imaging and diagnosis. The unique morphology of nanostars and their optical properties make them versatile candidates for multifunctional theranostic platforms, allowing for a synergistic approach to cancer treatment, including PDT, chemotherapy, and targeted drug delivery [168,224,225].

Quantum dots, specifically graphene and carbon quantum dots, are utilized in cancer therapy due to their distinctive features. These nanoscale semiconductor particles offer size-tunable fluorescence and high drug-loading capacity, making them ideal for high-resolution fluorescence imaging and drug delivery [226,227]. Additionally, when engineered appropriately, their biocompatibility reduces potential cytotoxicity, enhancing their suitability for in vivo applications [228]. Quantum dots can be functionalized with targeting ligands, such as folic acid, to improve specificity for cancer cells, minimizing damage to healthy tissues [227,229]. Furthermore, the pH-responsive behavior of some quantum dots ensures controlled and targeted drug release in an acidic tumor microenvironment [228].

In the realm of prostate cancer therapy, a distinct approach employs a tumor microenvironment-activated nanoprobe platform, enabling targeted delivery and controlled release of antitumor drugs for castration-resistant prostate cancer [230]. A dual-fluorescent nanoprobe designed for tumor detection and diagnosis is also reported, showcasing stability and high fluorescence intensity [231]. For hepatocellular carcinoma, a redox-responsive theranostic nanoprobe is developed [232]. Furthermore, a study presents HA-mediated multifunctional Fe_3_O_4_ nanoparticles for early and precise detection of pancreatic cancer, combining targeted delivery and T2-weighted magnetic resonance imaging [233]. In oral squamous cell carcinoma (OSCC), a multimodal NIR-II probe is designed for diagnosis and therapy, offering imaging capability and anti-tumor efficacy [234]. Another study introduces a multimodal nanoprobe combining photothermal therapy with chemotherapy to detect OSCC tumors and metastatic lymph nodes [235]. A unique development focuses on a single-cell sensor with a spatial architecture for high-precision single-cell analysis [29]. Finally, a theranostic fluorescent nanoprobe is designed for hyaluronidase detection, self-targeted imaging, and drug delivery, showcasing the great potential for cancer theranostics [236].

#### 3.5.4 Trends in delivery and therapy methods

Hypoxia is a consequence of the rapid and irregular growth of cancer cells, leading to the inability of a blood vessel to provide adequate oxygen supply to an entire tumor tissue [237]. This situation triggers adaptive responses at a cellular level, particularly by activating hypoxia-inducible factor-1 (HIF-1), which plays a central role in responding to and coping with hypoxic conditions [238]. Activation of HIF-1 leads to several biological changes that support the survival of cancer cells under low oxygen pressure. This includes stimulation of angiogenesis where HIF-1 triggers the production of blood vessel growth factors such as vascular endothelial growth factor (VEGF) to promote the formation of new blood vessels [239]. Moreover, HIF-1 also stimulates glycolysis, an oxygen-independent metabolic process, to ensure an adequate energy supply. HIF-1 activation can also contribute to therapy resistance, helping cancer cells survive and thrive in an unsupportive environment [240].

Therefore, researchers use strategies that involve targeting hypoxia as a vulnerability in cancer cell growth (see Fig. 11D). Most studies employ a hypoxic conditioning approach, where hypoxic conditions are triggered or manipulated to enhance the sensitivity of cancer cells to specific therapies [208,241]. Some studies utilize nanoparticles as carriers of active substances in the tumor microenvironment with hypoxia sensitivity [[242], [243], [244]]. These nanoparticles are designed to respond to hypoxic conditions, such as enhancing drug release or providing an environment that supports specific therapies. These studies often leverage specific receptors or cellular characteristics of cancer cells to direct nanoparticles to a target, increasing accumulation within the tumor.

Oxygen sensitization in hypoxic tumor conditions provides additional resistance to therapies, especially radiotherapy, which requires oxygen for its therapeutic effects [99,241,245]. Increasing oxygen availability in the tumor environment, either through oxygen release from nanoparticles or enhanced vascularization, can enhance the sensitivity of cancer cells to radiotherapy [243]. Inhibiting HIF-1, which increases in hypoxic conditions, can reduce pro-survival responses and enhance therapeutic effects [246]. Combination therapy approaches, such as using PDT and hypoxic conditioning, create a synergistic effect by exploiting hypoxic conditions to stimulate photochemical reactions, enhancing overall therapeutic responses [247].

Using pH-responsive drug delivery systems in cancer therapy has also emerged as a significant trend, providing a strategically advantageous approach for specific and effective treatment. These systems are designed to respond to changes in pH within the tumor microenvironment, which tends to be more acidic than normal tissues. The advantage of this approach lies in its ability to selectively trigger drug release at a tumor site, extending to specific cellular organelles such as lysosomes and endosomes [[248], [249], [250]]. This dual pH-responsive strategy acts as a targeted delivery and facilitates controlled release by responding to pH changes in a gradient manner [248]. Unlike relying on a single material such as KLA peptide for mitochondria targeting, this method allows using various materials sensitive to the pH of mitochondria, such as triphenylphosphonium (TPP) [251]. In contrast to receptor-targeted conjugates, which are limited in number, this approach can overcome resistance by switching to another pH-responsive material [214].

Some studies commonly perform co-delivery for dual therapies such as dual-chemotherapy, photo-chemotherapy, radio-chemotherapy, sonophototherapy, or other possible combinations. One study aimed to inhibit tumor development by simultaneously targeting an interaction between cancer-associated fibroblasts (CAFs) and tumor cells. They developed a pH-responsive nanoparticle (MIF/DOX-sul-HA NPs) based on sulfated HA polymer (sul-HA) for co-delivery of DOX and mifepristone (MIF). Results showed a stronger anti-proliferative and anti-metastatic effect compared to single-free drugs [73]. Another study addressed triple-negative breast cancer by designing a liposome modified with HA loaded with cisplatin and hesperetin for active targeting. This co-delivery system enhanced deep tumor penetration and inhibited the PI3K/Akt/mTOR pathway, demonstrating effective anti-tumor and anti-metastatic capabilities in triple-negative breast cancer with minimal side effects [252]. Similarly, another work developed chitosan-based nanoparticles modified with beta-cyclodextrin for co-delivering small interfering RNA MDR1 and DOX, achieving synergistic drug delivery with simultaneous control over a release of two different therapeutic agents [253]. Another study highlights a self-amplifying prodrug/photosensitizer activation positive feedback loop and demonstrates a dual-activated "low toxic-to-toxic" transformable treatment pattern for tumor-specific chemo-PDT [254].

#### 3.5.5 Trends in diagnostics

Ultrasonography or ultrasound imaging is a medical imaging technique that utilizes high-frequency sound waves to generate images of the human body's internal structures [255]. In this process, a transducer is used to transmit sound waves into the body and reflected signals are analyzed to visually represent a specific organ or area. The primary advantages of ultrasound imaging in cancer diagnosis are its non-invasiveness and lack of ionizing radiation, making it safe for repeated use [256]. Furthermore, real-time imaging capabilities allow direct observation of structural movement and blood flow [257]. This technique can also differentiate soft tissues, aiding in identifying tumors or cysts. The cost-effectiveness and guidance capabilities for invasive procedures, such as biopsies, make ultrasound an economical choice [258].

Tao S. et al. (2022) explored the use of sonotherapy to enhance the penetration of HA polymer conjugates with camptothecin and DOX into the brain, specifically in glioblastoma multiforme (GBM), with synergistic outcomes influenced by the drug ratio in the polymer nanocomplex [75]. Lisheng Z. et al. (2022) developed ultrasound and near-infrared (NIR)-responsive nanocapsules loaded with indocyanine green (ICG) and small gold nanoparticles (TAuNCs) for sonophotothermal therapy, demonstrating the potential of combining nanoparticles and ultrasound in breast cancer treatment [259]. Another study reported a low-frequency ultrasound (LFUS)-responsive pegylated liposome-based drug delivery platform, showing the potential application of combined nanoparticle and ultrasound therapy in breast cancer treatment [260]. Qianhua F. et al. (2017) introduced a pH/ultrasound-responsive gas generator based on mesoporous calcium carbonate nanoparticles (MCC) and HA, demonstrating a potential application of sonotherapy to induce therapeutic effects at the cellular level, resulting in cavitation-mediated irreversible cell necrosis. Additionally, this approach led to the destruction of blood vessels, further obstructing a blood supply and inducing a 'bystander effect' [261]. Hongyun Z. et al. (2018) developed a multifunctional ultrasonic molecular probe using a phase-change lipid nanoparticle-mediated by HA and cell-penetrating peptide for theranostics in hepatocellular carcinoma (HCC) [262].

X-ray imaging is crucial in diagnosing and tracking nanoparticles for cancer research. Various nanoparticles, such as BiOI, gadolinium oxide, and Bi_2_Se_3_, are synthesized and coated with hyaluronic acid (HA) for tumor targeting and diagnosis. One study developed BiOI nanoparticles coated with HA to enhance the targeting of tumor cells with CD44 overexpression. X-ray CT was employed to assess the contrast and effectiveness of BiOI as a contrast agent [263]. Another multifunctional nanoplatform based on Au nanocage-HA was developed for photoacoustic (PA) imaging, photothermal therapy (PTT), radiotherapy, and photodynamic therapy (PDT), with X-ray imaging serving as a tool to evaluate tumor growth and the effectiveness of combination therapy [264]. Gd_2_O_3_ nanoparticles coated with BSA and HA were also explored for tumor targeting and redox-responsive drug release, with X-ray CT as a visualization method to evaluate a tumor uptake level [265].

In another study, a bimodal tumor-targeted nanoplatform with Bi_2_Se_3_ HNCs-HA/GA was developed for low-temperature photothermal-radio combined therapy (PTT RT), where X-ray imaging was utilized to guide therapy and assess therapeutic effects at the cellular level [266]. Another nanoplatform, Bi_2_Se_3_@HA-doped PPy/ZnPc, was designed for multimodal imaging-guided phototherapy, with X-ray imaging providing contrast and identifying tumor locations [267]. A drug delivery system targeting CD44 and N-cadherin was developed using a mesoporous titanium dioxide nanoparticle (MTN)-based PDT, with X-ray imaging acting as a trigger for ROS production and therapeutic effect evaluation [268]. Fe_3_O_4_/Au-HA nanoparticle was developed for dual-mode tumor MR and CT imaging, and X-ray imaging was used to detect and visualize tumors with CD44 overexpression [224]. Another study evaluated HA-DESPIONs for radiosensitivity and hyperthermia response in CD44-expressing cancer cells, with X-ray imaging as a primary tool to observe therapeutic effects at a cellular level [46]. Finally, TaOxNPs nanoparticles were developed for fluorescence/X-ray CT imaging and pH-responsive drug delivery, with X-ray imaging serving as a method for monitoring and visualizing tumors and assessing overall therapeutic effectiveness [269].

In cancer therapy, nanoprobe refers to nanoscale technology with potential applications in diagnostics and therapeutics. In the diagnostic context, nanoprobes can serve as imaging agents to detect cancer cells or molecular changes at the cellular level. They can be utilized in imaging techniques such as MRI, CT, or positron emission tomography (PET) to provide accurate insights into the location and characteristics of tumors [270]. Alternatively, nanoprobes can function as cancer biomarker detection tools, offering rapid and precise information about the presence of the disease. In the therapeutic context, nanoprobes can act as drug delivery systems, delivering therapeutic agents directly to affected areas and minimizing side effects on surrounding healthy cells [271]. They can also play a role in thermal therapy, generating heat to damage cancer cells, or in PDT, where they are activated by light to induce chemical reactions that kill cancer cells [272]. Several studies have developed innovative nanoprobe systems for cancer diagnosis and therapy. In one study, a dual-targeting composite nanoprobe enhances sentinel lymph node imaging for breast tumor staging [273]. Another investigation introduces a photo-controlled nanodrug that effectively halts macrophage polarization in cancer and age-related macular degeneration [273]. Utilizing porous Fe_3_O_4_ nanoparticles, a different nanoprobe system demonstrates targeted combined photothermal/photodynamic therapy for lung cancer [274].

## 4 Limitation

NER algorithm utilizes SciSpacy package with a large version database. However, it does not include sufficient data on polymers. This is evidenced by several synthetic polymers not being recognized, requiring manual addition to the dataset. Some terms in material sciences may not be included, resulting in their failure to be recognized during the extraction process of keywords/word tokens. Abbreviations used in several articles may not be recognized within the SciSpacy dataset.

## 5 Author perspective

The future of HA-based nanoparticle research holds exciting possibilities, but several key areas need deeper exploration. One particularly compelling direction is the dual-targeting and dual-responsive systems using HA conjugates. While IL-15 and tetrac show great potential, their therapeutic use remains underexplored, especially in complex tumor environments. Further research should focus on how these novel APIs can work within advanced delivery platforms to overcome drug resistance and enhance targeted drug release.

Another area that excites us is the synergy between photodynamic and photothermal therapies. Both approaches have shown promising results, particularly in breast cancer treatment, but there's a strong case for expanding their application to more aggressive and treatment-resistant cancers. We believe combining these therapies with HA-based nanoparticles, which offer precise drug delivery and improved patient outcomes, will be critical to advancing cancer treatments. Theranostic platforms also present a fascinating frontier. The integration of diagnosis and therapy in a single nanoprobe is something that could revolutionize cancer treatment. Advances in nanoprobe technologies, such as quantum dots and nanostars, offer great potential for enhanced imaging and targeted drug delivery, particularly in identifying metastatic tumors. However, stability and biocompatibility remain challenges, and these must be addressed for real clinical impact.

As we transition from preclinical insights to clinical application, the role of clinical trials becomes paramount in establishing the safety and efficacy of conjugated hyaluronic acid (HA)-based nanoparticles for cancer therapy. Currently, there are 67 clinical trials related to using hyaluronic acid in cancer therapy [275]. None of these trials focus specifically on conjugated HA-based nanoparticles, particularly in their dual targeting and co-delivery application, which are more effective and safer in preclinical studies. These trials not only assess therapeutic outcomes but also address critical regulatory considerations that ensure these innovative treatments meet stringent safety standards before reaching patients. Importantly, a patient-centric approach is essential, as it emphasizes tailoring therapies to individual needs and responses, thereby enhancing treatment effectiveness and minimizing adverse effects.

## 6 Conclusion

In conclusion, the bibliometric analysis, incorporating techniques such as NER, LDA, and conventional bibliometric tools, revealed prominent trends in developing HA-based nanoparticles for cancer therapy. Our investigation identified key drug entities, which are DOX, siRNA, PTX, and CUR. It highlighted major themes, including surface modification for specific targeting, strategies for enhancing apoptosis and toxicity, controlled drug release mechanisms, and diverse drug delivery methods. A shift in research focus from optimizing substance toxicity to specific targeting strategies and precise release mechanisms is evident. Influential modifiers like adamantane and protamine contributed to nanoparticle systems' structural flexibility and adjuvant effects. Noteworthy conjugates, including dextran, streptavidin, and catechol, played vital roles in stabilizing nanoparticles and enhancing drug or metal loading. Diverse nanoparticle morphology, such as nanostars and quantum dots, showcased versatility in multifunctional theranostic platforms. Trends in delivery and therapy methods highlighted strategies to address challenges like hypoxia in the tumor microenvironment and the development of pH-responsive drug delivery systems. Additionally, a shift towards combination therapies gained prevalence. Diagnostic advancements were evident through ultrasound and X-ray imaging, emphasizing their roles in guiding therapy and assessing cellular-level therapeutic effects. Nanoprobes emerged as versatile tools with dual functionality, serving as imaging agents and drug delivery systems for precise cancer diagnosis and treatment. These findings reflect the dynamic landscape of HA-based nanoparticle research in cancer therapy, emphasizing advancements in targeted drug delivery, therapeutic efficacy, and multimodal diagnostic approaches to improve overall patient outcomes.

## CRediT authorship contribution statement

**Abd Kakhar Umar:** Writing – review & editing, Writing – original draft, Visualization, Validation, Supervision, Software, Resources, Project administration, Methodology, Investigation, Formal analysis, Data curation, Conceptualization. **Patanachai K. Limpikirati:** Writing – review & editing, Writing – original draft, Validation, Supervision, Resources, Investigation, Formal analysis, Data curation, Conceptualization. **Bachtiar Rivai:** Writing – review & editing, Writing – original draft, Visualization, Investigation, Formal analysis, Data curation, Conceptualization. **Ilham Ardiansah:** Writing – review & editing, Writing – original draft, Visualization, Software, Resources, Methodology, Investigation, Formal analysis, Data curation. **Sriwidodo Sriwidodo:** Writing – review & editing, Writing – original draft, Visualization, Investigation, Formal analysis. **Jittima Amie Luckanagul:** Writing – review & editing, Writing – original draft, Visualization, Validation, Supervision, Project administration, Investigation, Funding acquisition, Formal analysis, Data curation, Conceptualization.

## Data availability

The data is available upon request to the first or corresponding author.

## Funding information

This study was supported by Program Management Unit for Human Resources & Institutional Development, Research and Innovation (PMU-B) (grant number B13F660137)

## Declaration of competing interest

The authors declare the following financial interests/personal relationships which may be considered as potential competing interests: Jittima Amie Luckanagul reports financial support was provided by 10.13039/501100002873Chulalongkorn University. If there are other authors, they declare that they have no known competing financial interests or personal relationships that could have appeared to influence the work reported in this paper.

## References

[1] Thomas H.F. (1978). Cancer and the environment. Environ. Health..

[2] Parsa N. (2012). Environmental factors inducing human cancers. Iran. J. Public Health.

[3] Kciuk M., Gielecińska A., Mujwar S., Kołat D., Kałuzińska-Kołat Ż., Celik I. (2023 Feb 19). Doxorubicin—an agent with multiple mechanisms of anticancer activity. Cells.

[4] Sharifi-Rad J., Quispe C., Patra J.K., Singh Y.D., Panda M.K., Das G., De Oliveira F.L. (2021 Oct 18). Paclitaxel: application in modern oncology and nanomedicine-based cancer therapy. Oxid. Med. Cell. Longev..

[5] Tacar O., Sriamornsak P., Dass C.R. (2012 Dec 26). Doxorubicin: an update on anticancer molecular action, toxicity and novel drug delivery systems. J. Pharm. Pharmacol..

[6] Li Y., Hou H., Zhang P., Zhang Z. (2020 Jan 1). Co-delivery of doxorubicin and paclitaxel by reduction/pH dual responsive nanocarriers for osteosarcoma therapy. Drug Deliv..

[7] Ioele G., Chieffallo M., Occhiuzzi M.A., De Luca M., Garofalo A., Ragno G. (2022 Aug 25). Anticancer drugs: recent strategies to improve stability profile, pharmacokinetic and pharmacodynamic properties. Molecules.

[8] Harris E.N., Baker E. (2020 May 15). Role of the hyaluronan receptor, stabilin-2/HARE, in health and disease. Int. J. Mol. Sci..

[9] Wu M., Du Y., Liu Y., He Y., Yang C., Wang W. (2014 Mar 25). Low molecular weight hyaluronan induces lymphangiogenesis through LYVE-1-mediated signaling pathways. Addison CL. PLoS One.

[10] Wolf K.J., Kumar S. (2019 Aug 12). Hyaluronic acid: incorporating the bio into the material. ACS Biomater. Sci. Eng..

[11] Kesharwani P., Chadar R., Sheikh A., Rizg W.Y., Safhi A.Y. (2022 Mar 31). CD44-Targeted nanocarrier for cancer therapy. Front. Pharmacol..

[12] Miyazaki M., Yuba E., Hayashi H., Harada A., Kono K. (2019 Nov 11). Development of pH-responsive hyaluronic acid-based antigen carriers for induction of antigen-specific cellular immune responses. ACS Biomater. Sci. Eng..

[13] Shahbazi M.A., Sedighi M., Bauleth-Ramos T., Kant K., Correia A., Poursina N. (2018 Dec 31). Targeted reinforcement of macrophage reprogramming toward M2 polarization by IL-4-loaded hyaluronic acid particles. ACS Omega.

[14] Nemec S., Ganda S., Al Taief K., Kopecky C., Kuchel R., Lebhar H. (2022 Oct 17). A tunable tumor microenvironment through recombinant bacterial collagen-hyaluronic acid hydrogels. ACS Appl. Bio Mater..

[15] Haas S., Hain N., Raoufi M., Handschuh-Wang S., Wang T., Jiang X. (2015 Mar 9). Enzyme degradable polymersomes from hyaluronic acid- block -poly(ε-caprolactone) copolymers for the detection of enzymes of pathogenic bacteria. Biomacromolecules.

[16] Choi S., Lee S.H., Park S., Park S.H., Park C., Key J. (2021). Indocyanine green-loaded PLGA nanoparticles conjugated with hyaluronic acid improve target specificity in cervical cancer tumors. Yonsei Med. J..

[17] Yang Y., Hu D., Lu Y., Chu B., He X., Chen Y. (2022 Jun). Tumor-targeted/reduction-triggered composite multifunctional nanoparticles for breast cancer chemo-photothermal combinational therapy. Acta Pharm. Sin. B.

[18] Cai L., Dong L., Sha X., Zhang S., Liu S., Song X. (2021 Nov). Exfoliation and in situ functionalization of MoS2 nanosheets for MRI-guided combined low-temperature photothermal therapy and chemotherapy. Mater. Des..

[19] Yoon H.M., Kang M.S., Choi G.E., Kim Y.J., Bae C.H., Yu Y.B. (2021 Dec 6). Stimuli-responsive drug delivery of doxorubicin using magnetic nanoparticle conjugated poly(ethylene glycol)-g-Chitosan copolymer. Int. J. Mol. Sci..

[20] Yuan Y., Liu J., Yu X., Liu X., Cheng Y., Zhou C. (2021 Nov). Tumor-targeting pH/redox dual-responsive nanosystem epigenetically reverses cancer drug resistance by co-delivering doxorubicin and GCN5 siRNA. Acta Biomater..

[21] Wang X., Xiong T., Cui M., Li N., Li Q., Zhu L. (2021 Dec 23). A novel targeted co-delivery nanosystem for enhanced ovarian cancer treatment via multidrug resistance reversion and mTOR-mediated signaling pathway. J Nanobiotechnology.

[22] Zeng X., Zhang Y., Xu X., Chen Z., Ma L., Wang Y. (2022 Dec 31). Construction of pH-sensitive targeted micelle system co-delivery with curcumin and dasatinib and evaluation of anti-liver cancer. Drug Deliv..

[23] Lu J., Luo B., Chen Z., Yuan Y., Kuang Y., Wan L. (2020 Mar). Host-guest fabrication of dual-responsive hyaluronic acid/mesoporous silica nanoparticle based drug delivery system for targeted cancer therapy. Int. J. Biol. Macromol..

[24] Liang J., Yang X., Liu D., Cong M., Song Y., Bai S. (2020 Aug 14). Lipid/hyaluronic acid–coated doxorubicin-Fe3O4 as a dual-targeting nanoparticle for enhanced cancer therapy. AAPS PharmSciTech.

[25] Hintze V., Schnabelrauch M., Rother S. (2022 Feb 11). Chemical modification of hyaluronan and their biomedical applications. Front. Chem..

[26] Mihajlovic M., Fermin L., Ito K., van Nostrum C.F., Vermonden T. (2021 Sep 1). Hyaluronic acid-based supramolecular hydrogels for biomedical applications. Multifunct Mater.

[27] Bergman K., Elvingson C., Hilborn J., Svensk G., Bowden T. (2007 Jul 1). Hyaluronic acid derivatives prepared in aqueous media by triazine-activated amidation. Biomacromolecules.

[28] Li J., Qiao M., Ji Y., Lin L., Zhang X., Linhardt R.J. (2020 Jun). Chemical, enzymatic and biological synthesis of hyaluronic acids. Int. J. Biol. Macromol..

[29] Long D., Chen C., Cui C., Yao Z., Yang P. (2018). A high precision MUA-spaced single-cell sensor for cellular receptor assay based on bifunctional Au@Cu-PbCQD nanoprobes. Nanoscale.

[30] Liu J., Zheng J., Nie H., Zhang D., Cao D., Xing Z. (2019 Jul). Molybdenum disulfide-based hyaluronic acid-guided multifunctional theranostic nanoplatform for magnetic resonance imaging and synergetic chemo-photothermal therapy. J. Colloid Interface Sci..

[31] Zhou J., Wang M., Han Y., Lai J., Chen J. (2020 Jan 21). Multistage-targeted gold/mesoporous silica nanocomposite hydrogel as in situ injectable drug release system for chemophotothermal synergistic cancer therapy. ACS Appl. Bio Mater..

[32] Sakurai Y., Kato A., Hida Y., Hamada J., Maishi N., Hida K. (2019 Oct). Synergistic enhancement of cellular uptake with CD44-expressing malignant pleural mesothelioma by combining cationic liposome and hyaluronic acid–lipid conjugate. J Pharm Sci.

[33] Zhou M., Lai W., Li G., Wang F., Liu W., Liao J. (2021 Jun 9). Platelet membrane-coated and VAR2CSA malaria protein-functionalized nanoparticles for targeted treatment of primary and metastatic cancer. ACS Appl. Mater. Interfaces.

[34] Liu J., Ma W., Kou W., Shang L., Huang R., Zhao J. (2019 Dec 20). Poly-amino acids coated gold nanorod and doxorubicin for synergistic photodynamic therapy and chemotherapy in ovarian cancer cells. Biosci. Rep..

[35] Yan J., Shan C., Liang C., Han J., He B., Sun Y. (2022 Dec 12). Smart multistage “trojan horse”-inspired bovine serum albumin-coated liposomes for enhancing tumor penetration and antitumor efficacy. Biomacromolecules.

[36] Pang J., Xing H., Sun Y., Feng S., Wang S. (2020 May). Non-small cell lung cancer combination therapy: hyaluronic acid modified, epidermal growth factor receptor targeted, pH sensitive lipid-polymer hybrid nanoparticles for the delivery of erlotinib plus bevacizumab. Biomed. Pharmacother..

[37] Liu Z., Chen H., Lv F., Wang J., Zhao S., Li Y. (2021 Aug 16). Sequential release of paclitaxel and imatinib from core–shell microparticles prepared by coaxial electrospray for vaginal therapy of cervical cancer. Int. J. Mol. Sci..

[38] Yang X., Shang P., Ji J., Malichewe C., Yao Z., Liao J. (2022 Jan 3). Hyaluronic acid-modified nanoparticles self-assembled from linoleic acid-conjugated chitosan for the codelivery of miR34a and doxorubicin in resistant breast cancer. Mol. Pharm..

[39] Chen G., Wei P., Huang L., Lei M., Zhang M., Lei J. (2021 Nov 6). pH-responsive hyaluronic acid nanoparticles codelivering DOX and ICG for effectively chemo-photothermal combination therapy. J. Nanoparticle Res..

[40] Wu H.C., Kuo W.T. (2021 Dec 13). Redox/pH-Responsive 2-in-1 chimeric nanoparticles for the Co-delivery of doxorubicin and siRNA. Polymers.

[41] Bastaki S., Aravindhan S., Ahmadpour Saheb N., Afsari Kashani M., Evgenievich Dorofeev A., Karoon Kiani F. (2021 Feb). Codelivery of STAT3 and PD-L1 siRNA by hyaluronate-TAT trimethyl/thiolated chitosan nanoparticles suppresses cancer progression in tumor-bearing mice. Life Sci..

[42] Hsiao K., Wu Y.J., Liu Z., Chuang C., Huang H., Kuo S. (2016 Mar 2). Anticancer effects of sinulariolide-conjugated hyaluronan nanoparticles on lung adenocarcinoma cells. Molecules.

[43] Ivashchenko O., Przysiecka Ł., Peplińska B., Jarek M., Coy E., Jurga S. (2018 Sep 5). Gel with silver and ultrasmall iron oxide nanoparticles produced with Amanita muscaria extract: physicochemical characterization, microstructure analysis and anticancer properties. Sci. Rep..

[44] Suksiriworapong J., Pongprasert N., Bunsupa S., Taresco V., Crucitti V.C., Janurai T. (2023 Jun 20). CD44-Targeted lipid polymer hybrid nanoparticles enhance anti-breast cancer effect of cordyceps militaris extracts. Pharmaceutics.

[45] Ran R., Liu Y., Gao H., Kuang Q., Zhang Q., Tang J. (2014 Dec). Enhanced gene delivery efficiency of cationic liposomes coated with PEGylated hyaluronic acid for anti P-glycoprotein siRNA: a potential candidate for overcoming multi-drug resistance. Int J Pharm.

[46] Thapa R., Galoforo S., Kandel S.M., El-dakdouki M.H., Wilson T.G., Huang X. (2015). Radiosensitizing and hyperthermic properties of hyaluronan conjugated, dextran-coated ferric oxide nanoparticles: implications for cancer stem cell therapy. J. Nanomater..

[47] Zhu L., Zhao Y., Liu T., Chen M., Qian W.P., Jiang B. (2022 Nov 22). Inhibition of NADPH oxidase-ROS signal using hyaluronic acid nanoparticles for overcoming radioresistance in cancer therapy. ACS Nano.

[48] Hu Q., Li H., Archibong E., Chen Q., Ruan H., Ahn S. (2021 Apr 26). Inhibition of post-surgery tumour recurrence via a hydrogel releasing CAR-T cells and anti-PDL1-conjugated platelets. Nat. Biomed. Eng..

[49] Liang Y., Li M., Huang Y., Guo B. (2021 Sep 11). An integrated strategy for rapid hemostasis during tumor resection and prevention of postoperative tumor recurrence of hepatocellular carcinoma by antibacterial shape memory cryogel. Small.

[50] Zhou H., Sun J., Wu J., Wei H., Zhou X. (2021 Apr). Biodegradable nanosonosensitizers with the multiple modulation of tumor microenvironment for enhanced sonodynamic therapy. Int J Nanomedicine.

[51] Xie R., Lian S., Peng H., OuYang C., Li S., Lu Y. (2019 May 6). Mitochondria and nuclei dual-targeted hollow carbon nanospheres for cancer chemophotodynamic synergistic therapy. Mol. Pharm..

[52] Machado V., Morais M., Medeiros R. (2022 Sep 30). Hyaluronic acid-based nanomaterials applied to cancer: where are we now?. Pharmaceutics.

[53] Jia Y., Chen S., Wang C., Sun T., Yang L. (2022 Aug 24). Hyaluronic acid-based nano drug delivery systems for breast cancer treatment: recent advances. Front. Bioeng. Biotechnol..

[54] Kim J., Moon M., Kim D., Heo S., Jeong Y. (2018 Oct 12). Hyaluronic acid-based nanomaterials for cancer therapy. Polymers.

[55] Fu C.P., Cai X.Y., Chen S.L., Yu H.W., Fang Y., Feng X.C. (2023 May 16). Hyaluronic acid-based nanocarriers for anticancer drug delivery. Polymers.

[56] Brito A.C.M., Oliveira M.C.F., Oliveira O.N., Silva F.N., Amancio D.R. (2023 Jun 14). Network analysis and natural language processing to obtain a landscape of the scientific literature on materials applications. ACS Appl. Mater. Interfaces.

[57] Brito A.C.M., Oliveira M.C.F., Oliveira O.N., Silva F.N., Amancio D.R. (2024 Jan 9). History of chemistry of materials according to topic evolution based on network analysis and natural language processing. Chem. Mater..

[58] Neumann M., King D., Beltagy I., Ammar W. (2019). Proceedings of the 18th BioNLP Workshop and Shared Task.

[59] Hu C., Gong H., He Y., Margherita A. (2022 Oct 12). Data driven identification of international cutting edge science and technologies using SpaCy. PLoS One.

[60] Campbell J.C., Hindle A., Stroulia E. (2015). Latent dirichlet allocation: extracting topics from software engineering data. Art Sci Anal Softw Data.

[61] Farkhod A., Abdusalomov A., Makhmudov F., Cho Y.I. (2021 Nov 23). LDA-based topic modeling sentiment analysis using topic/document/sentence (TDS) model. Appl. Sci..

[62] Specht A., Crowston K., Lozano S. (2022 Nov 29). Interdisciplinary collaboration from diverse science teams can produce significant outcomes. PLoS One.

[63] Nane T. (2000). Time to first citation estimation in the presence of additional information. Proc ISSI 2015 Istanbul 15th Int Soc Sci Inf Conf..

[64] Keathley-Herring H., Van Aken E., Gonzalez-Aleu F., Deschamps F., Letens G., Orlandini P.C. (2016 Nov 13). Assessing the maturity of a research area: bibliometric review and proposed framework. Scientometrics.

[65] Keathley H., Gonzalez Aleu F., Cárdenas Orlandini P.F., Van Aken E., Deschamps F., Rosa Leite L. (2013). Proposed maturity assessment framework for a research field. IIE Annu Conf Expo.

[66] Shah F.A., Jawaid S.A. (2023 Jan 24). The h-index: an indicator of research and publication output. Pakistan J. Med. Sci..

[67] Xie Y., Zhang C., Lai Q. (2014 Jul 16). China's rise as a major contributor to science and technology. Proc Natl Acad Sci.

[68] Jelodar H., Wang Y., Yuan C., Feng X., Jiang X., Li Y. (2019 Jun 28). Latent Dirichlet allocation (LDA) and topic modeling: models, applications, a survey. Multimed Tools Appl.

[69] Asmussen C.B., Møller C. (2019 Dec 19). Smart literature review: a practical topic modelling approach to exploratory literature review. J Big Data.

[70] He F., Xie C., Xu X. (2023 Aug). Hyaluronic acid-modified yeast β-glucan particles delivering doxorubicin for treatment of breast cancer. Carbohydr. Polym..

[71] Çağdaş Tunalı B., Çelik E., Budak Yıldıran F.A., Türk M. (2023 Apr 27). Delivery of <scp>siRNA</scp> using hyaluronic acid‐guided nanoparticles for downregulation of <scp>CXCR4</scp&gt. Biopolymers.

[72] Kou Q., Huang Y., Su Y., Lu L., Li X., Jiang H. (2023). Erythrocyte membrane-camouflaged DNA-functionalized upconversion nanoparticles for tumor-targeted chemotherapy and immunotherapy. Nanoscale.

[73] Wang D., Wu J., Qi C., Dong J., Ding X., Yu G. (2023 May). pH-responsive sulfated hyaluronic acid nanoparticles targeting tumor cells and CAFs for the treatment of breast cancer. Recent Pat. Anti-Cancer Drug Discov..

[74] Shao Y., Luo W., Guo Q., Li X., Zhang Q., Li J. (2019 Jun). In vitro and in vivo effect of hyaluronic acid modified, doxorubicin and gallic acid co-delivered lipid-polymeric hybrid nano-system for leukemia therapy. Drug Des Devel Ther.

[75] Sun T., Krishnan V., Pan D.C., Filippov S.K., Ravid S., Sarode A. (2023 Mar 19). Ultrasound‐mediated delivery of flexibility‐tunable polymer drug conjugates for treating glioblastoma. Bioeng Transl Med.

[76] Hayward S.L., Wilson C.L., Kidambi S. (2016 Jun 7). Hyaluronic acid-conjugated liposome nanoparticles for targeted delivery to CD44 overexpressing glioblastoma cells. Oncotarget.

[77] Li Y., Ertas Y.N., Jafari A., Taheri M., Pilehvar Y. (2023 Sep). Co-delivery of curcumin and chrysin through pH-sensitive hyaluronan-modified hollow mesoporous silica nanoparticles for enhanced synergistic anticancer efficiency against thyroid cancer cells. J. Drug Deliv. Sci. Technol..

[78] Xu X., Sabanayagam C.R., Harrington D.A., Farach-Carson M.C., Jia X. (2014 Mar). A hydrogel-based tumor model for the evaluation of nanoparticle-based cancer therapeutics. Biomaterials.

[79] Li K., Zhan W., Chen Y., Jha R.K., Chen X. (2019 Dec 18). Docetaxel and doxorubicin codelivery by nanocarriers for synergistic treatment of prostate cancer. Front. Pharmacol..

[80] Li K., Zhan W., Jia M., Zhao Y., Liu Y., Jha R.K. (2020). Dual loading of nanoparticles with doxorubicin and icotinib for the synergistic suppression of non-small cell lung cancer. Int. J. Med. Sci..

[81] Lee R., Choi Y.J., Jeong M.S., Park Y Il, Motoyama K., Kim M.W. (2020 Mar 18). Hyaluronic acid-decorated glycol chitosan nanoparticles for pH-sensitive controlled release of doxorubicin and celecoxib in nonsmall cell lung cancer. Bioconjug Chem..

[82] Kong C.Y., Guo Z., Song P., Zhang X., Yuan Y.P., Teng T. (2022). Underlying the mechanisms of doxorubicin-induced acute cardiotoxicity: oxidative stress and cell death. Int. J. Biol. Sci..

[83] Sardão V.A., Oliveira P.J., Holy J., Oliveira C.R., Wallace K.B. (2009 Sep 30). Doxorubicin-induced mitochondrial dysfunction is secondary to nuclear p53 activation in H9c2 cardiomyoblasts. Cancer Chemother. Pharmacol..

[84] Bousset L., Gil J. (2022 Nov 23). Targeting senescence as an anticancer therapy. Mol Oncol [Internet].

[85] Lim P.T., Goh B.H., Lee W.L. (2022). Paclitaxel.

[86] Markman M., Mekhail T.M. (2002 Jun 25). Paclitaxel in cancer therapy. Expert Opin Pharmacother.

[87] Shi Y., Zhou M., Zhang Y., Wang Y., Cheng J. (2023 Mar). MRI-guided dual-responsive anti-tumor nanostructures for synergistic chemo-photothermal therapy and chemodynamic therapy. Acta Biomater..

[88] Deng X., Cao M., Zhang J., Hu K., Yin Z., Zhou Z. (2014 May). Hyaluronic acid-chitosan nanoparticles for co-delivery of MiR-34a and doxorubicin in therapy against triple negative breast cancer. Biomaterials.

[89] Huang P., Yang C., Liu J., Wang W., Guo S., Li J. (2014). Improving the oral delivery efficiency of anticancer drugs by chitosan coated polycaprolactone-grafted hyaluronic acid nanoparticles. J. Mater. Chem. B.

[90] Zhou Q., Wu J., Lian B., Zhang B., Zhang P., Pan R. (2022 Oct 28). Glycyrrhetinic acid-modified sulfated hyaluronic acid nanoparticles coencapsulating doxorubicin and magnolol for the synergistic treatment of hepatocellular carcinoma. ACS Appl. Nano Mater..

[91] Guo Q., Li J., Mao J., Chen W., Yang M., Yang Y. (2023 Nov 13). Hollow MIL-125 nanoparticles loading doxorubicin prodrug and 3-methyladenine for reversal of tumor multidrug resistance. J. Funct. Biomater..

[92] Choi K.Y., Silvestre O.F., Huang X., Min K.H., Howard G.P., Hida N. (2014 May 27). Versatile RNA interference nanoplatform for systemic delivery of RNAs. ACS Nano.

[93] Peng H., Qiao L., Shan G., Gao M., Zhang R., Yi X. (2022 Sep). Stepwise responsive carboxymethyl chitosan-based nanoplatform for effective drug-resistant breast cancer suppression. Carbohydr. Polym..

[94] Shen Y., Wang J., Li Y., Tian Y., Sun H., Ammar O. (2015). Co-delivery of siRNA and paclitaxel into cancer cells by hyaluronic acid modified redox-sensitive disulfide-crosslinked PLGA–PEI nanoparticles. RSC Adv..

[95] Hao Y., Gao Y., Wu Y., An C. (2018  Dec 3). The AIB1siRNA-loaded hyaluronic acid-assembled PEI/heparin/Ca2+ nanocomplex as a novel therapeutic strategy in lung cancer treatment. Int. J. Mol. Med..

[96] Subhan M.A., Torchilin V.P. (2019 Dec). Efficient nanocarriers of siRNA therapeutics for cancer treatment. Transl. Res..

[97] Yang X., Lyer A.K., Singh A., Choy E., Hornicek F.J., Amiji M.M. (2015 Feb 17). MDR1 siRNA loaded hyaluronic acid-based CD44 targeted nanoparticle systems circumvent paclitaxel resistance in ovarian cancer. Sci. Rep..

[98] Talekar M., Ouyang Q., Goldberg M.S., Amiji M.M. (2015 Jul 1). Cosilencing of PKM-2 and MDR-1 sensitizes multidrug-resistant ovarian cancer cells to paclitaxel in a murine model of ovarian cancer. Mol Cancer Ther.

[99] Li Y., Zhang J., Wang B., Shen Y., Ouahab A. (2016 May 3). Co-delivery of siRNA and hypericin into cancer cells by hyaluronic acid modified PLGA-PEI nanoparticles. Drug Dev. Ind. Pharm..

[100] Tian G., Pan R., Zhang B., Qu M., Lian B., Jiang H. (2019 Jan 22). Liver-targeted combination therapy basing on glycyrrhizic acid-modified DSPE-PEG-PEI nanoparticles for Co-delivery of doxorubicin and bcl-2 siRNA. Front. Pharmacol..

[101] Xu Z., Wang Y., Zhang L., Huang L. (2014 Apr 22). Nanoparticle-Delivered transforming growth factor-β siRNA enhances vaccination against advanced melanoma by modifying tumor microenvironment. ACS Nano.

[102] Shahidi M., Abazari O., Dayati P., Reza J.Z., Modarressi M.H., Tofighi D. (2023 Feb). Using chitosan-stabilized, hyaluronic acid-modified selenium nanoparticles to deliver CD44-targeted PLK1 siRNAs for treating bladder cancer. Nanomedicine..

[103] Arranja A.G., Pathak V., Lammers T., Shi Y. (2017 Jan). Tumor-targeted nanomedicines for cancer theranostics. Pharmacol. Res..

[104] Russo S. (2016). Gastrointestinal Cancers Symposium: update on pancreatic cancer. Ann. Gastroenterol..

[105] Tabernero J., Shapiro G.I., LoRusso P.M., Cervantes A., Schwartz G.K., Weiss G.J. (2013 Apr 1). First-in-Humans trial of an RNA interference therapeutic targeting VEGF and KSP in cancer patients with liver involvement. Cancer Discov..

[106] Jiang Y., Huo S., Hardie J., Liang X.J., Rotello V.M. (2016 Apr 2). Progress and perspective of inorganic nanoparticle-based siRNA delivery systems. Expert Opin Drug Deliv.

[107] Hwang H.J., Lee Y.R., Kang D., Lee H.C., Seo H.R., Ryu J.K. (2020 Oct). Endothelial cells under therapy-induced senescence secrete CXCL11, which increases aggressiveness of breast cancer cells. Cancer Lett..

[108] Hattab D., Gazzali A.M., Bakhtiar A. (2021 Jul 2). Clinical advances of siRNA-based nanotherapeutics for cancer treatment. Pharmaceutics.

[109] Ghosh S., Dutta S., Sarkar A., Kundu M., Sil P.C. (2021 Jan). Targeted delivery of curcumin in breast cancer cells via hyaluronic acid modified mesoporous silica nanoparticle to enhance anticancer efficiency. Colloids Surfaces B Biointerfaces.

[110] Adaileh F., Alshaer W., Nsairat H., Alqudah D.A., Wehaibi S., Daoud F. (2023 Sep). Curcumin-loaded γ -cyclodextrin-grafted hyaluronic acid nanoassimblies: in vitro investigation of anti-proliferative, wound healing, and anti-inflammatory potential. J. Drug Deliv. Sci. Technol..

[111] Rong L., Liu Y., Fan Y., Xiao J., Su Y., Lu L. (2023 Jun). Injectable nano-composite hydrogels based on hyaluronic acid-chitosan derivatives for simultaneous photothermal-chemo therapy of cancer with anti-inflammatory capacity. Carbohydr. Polym..

[112] Kumari M., Purohit M.P., Patnaik S., Shukla Y., Kumar P., Gupta K.C. (2018 Sep). Curcumin loaded selenium nanoparticles synergize the anticancer potential of doxorubicin contained in self-assembled, cell receptor targeted nanoparticles. Eur. J. Pharm. Biopharm..

[113] Wang J., Zhao H., Song W., Gu M., Liu Y., Liu B. (2022 Jul 4). Gold nanoparticle-decorated drug nanocrystals for enhancing anticancer efficacy and reversing drug resistance through chemo-/photothermal therapy. Mol. Pharm..

[114] Xie J., Wang H., Huang Q., Lin J., Wen H., Miao Y. (2023 Oct). Enhanced cytotoxicity to lung cancer cells by mitochondrial delivery of camptothecin. Eur J Pharm Sci.

[115] Kurien B.T., Scofield R.H. (2009 Jul). Increasing aqueous solubility of curcumin for improving bioavailability. Trends Pharmacol. Sci..

[116] Yang X., Zhao H., Lei H., Yuan B., Mao S., Xin M. (2021 Mar 18). Synthesis and biological evaluation of 10‐substituted camptothecin derivatives with improved water solubility and activity. ChemMedChem.

[117] Sharma R.A., Steward W.P., Gescher A.J. (2007). Pharmacokinetics and Pharmacodynamics of Curcumin.

[118] Kim Y.J., Kim Y.J. (2017). Pharmacokinetics and Enterohepatic Recycling of CZ48 , a Lactone-Stabilized Camptothecin : Effects of Nanosuspension Formulation.

[119] Wang Y.J., Pan M.H., Cheng A.L., Lin L.I., Ho Y.S., Hsieh C.Y. (1997 Aug). Stability of curcumin in buffer solutions and characterization of its degradation products. J. Pharm. Biomed. Anal..

[120] Bacherikov V.A. (2022 Dec). Total synthesis, mechanism of action, and antitumor efficacy of camptothecin and some of its analogues. Anti Cancer Agents Med. Chem..

[121] Zhang Y., Ranaei Pirmardan E., Barakat A., Naseri M., Hafezi-Moghadam A. (2022 Sep 28). Nanoarchitectonics for photo-controlled intracellular drug release in immune modulation. ACS Appl. Mater. Interfaces.

[122] Al-Serwi R.H., Eladl M.A., El-Sherbiny M., Saleh M.A., Othman G., Alshahrani S.M. (2023 May 17). Targeted drug administration onto cancer cells using hyaluronic acid–quercetin-conjugated silver nanoparticles. Molecules.

[123] Quan X.Q., Kang L., Yin X.Z., Jin Z.H., Gao Z.G. (2015 Jun). Synthesis of PEGylated hyaluronic acid for loading dichloro(1,2-diaminocyclohexane)platinum(II) (DACHPt) in nanoparticles for cancer treatment. Chinese Chem Lett.

[124] Li Y., Ruan S., Guo J., He Z., Xia Q., Wu T. (2022 Aug 1). B16F10 cell membrane-based nanovesicles for melanoma therapy are superior to hyaluronic acid-modified nanocarriers. Mol. Pharm..

[125] Salimifard S., Karoon Kiani F., Sadat Eshaghi F., Izadi S., Shahdadnejad K., Masjedi A. (2020 Nov). Codelivery of BV6 and anti-IL6 siRNA by hyaluronate-conjugated PEG-chitosan-lactate nanoparticles inhibits tumor progression. Life Sci..

[126] Wan S., Fan Q., Wu Y., Zhang J., Qiao G., Jiang N. (2023 Feb 13). Curcumin-loaded platelet membrane bioinspired chitosan-modified liposome for effective cancer therapy. Pharmaceutics.

[127] Gupta C., Singh P., Vaidya S., Ambre P., Coutinho E. (2023 Jul). A novel thermoresponsive nano carrier matrix of hyaluronic acid, methotrexate and chitosan to target the cluster of differentiation 44 receptors in tumors. Int. J. Biol. Macromol..

[128] Xiao J., Sun Q., Ran L., Wang Y., Qin X., Xu X. (2023 Feb 13). pH-responsive selenium nanoplatform for highly efficient cancer starvation therapy by atorvastatin delivery. ACS Biomater. Sci. Eng..

[129] Chen Y., Wang X., Lu Z., Chang C., Zhang Y., Lu B. (2023 Jul 3). Dual-responsive targeted hollow mesoporous silica nanoparticles for cancer photodynamic therapy and chemotherapy. J. Macromol. Sci. Part A..

[130] Masjedi A., Ahmadi A., Atyabi F., Farhadi S., Irandoust M., Khazaei-Poul Y. (2020 Apr). Silencing of IL-6 and STAT3 by siRNA loaded hyaluronate-N,N,N-trimethyl chitosan nanoparticles potently reduces cancer cell progression. Int. J. Biol. Macromol..

[131] Carvalho V.F.M., Salata G.C., de Matos J.K.R., Costa-Fernandez S., Chorilli M., Steiner A.A. (2019 Aug). Optimization of composition and obtainment parameters of biocompatible nanoemulsions intended for intraductal administration of piplartine (piperlongumine) and mammary tissue targeting. Int J Pharm.

[132] Sang M.M., Liu F.L., Wang Y., Luo R.J., Huan X.X., Han L.F. (2018 Jan 1). A novel redox/pH dual-responsive and hyaluronic acid-decorated multifunctional magnetic complex micelle for targeted gambogic acid delivery for the treatment of triple negative breast cancer. Drug Deliv..

[133] Sang M., Zhang Z., Liu F., Hu L., Li L., Chen L. (2018 Mar 1). Multifunctional hyaluronic acid-decorated redox-responsive magnetic complex micelle for targeted drug delivery with enhanced antitumor efficiency and anti-cell-migration activity. J. Biomed. Nanotechnol..

[134] Wu Y., Zhang X., Li H., Deng P., Li H., He T. (2018). A core/shell stabilized polysaccharide-based nanoparticle with intracellular environment-sensitive drug delivery for breast cancer therapy. J. Mater. Chem. B.

[135] Zhang R., Ru Y., Gao Y., Li J., Mao S. (2017 Sep). Layer-by-layer nanoparticles co-loading gemcitabine and platinum (IV) prodrugs for synergistic combination therapy of lung cancer. Drug Des Devel Ther.

[136] Ramasamy T., Tran T.H., Choi J.Y., Cho H.J., Kim J.H., Yong C.S. (2014 Feb). Layer-by-layer coated lipid–polymer hybrid nanoparticles designed for use in anticancer drug delivery. Carbohydr. Polym..

[137] Maiolino S., Russo A., Pagliara V., Conte C., Ungaro F., Russo G. (2015 Dec 3). Biodegradable nanoparticles sequentially decorated with Polyethyleneimine and Hyaluronan for the targeted delivery of docetaxel to airway cancer cells. J Nanobiotechnology.

[138] Chen H.A., Lu Y.J., Dash B.S., Chao Y.K., Chen J.P. (2023 Jan 14). Hyaluronic acid-modified cisplatin-encapsulated poly(lactic-co-glycolic acid) magnetic nanoparticles for dual-targeted NIR-responsive chemo-photothermal combination cancer therapy. Pharmaceutics.

[139] Huang W.C., Chen S.H., Chiang W.H., Huang C.W., Lo C.L., Chern C.S. (2016 Dec 12). Tumor microenvironment-responsive nanoparticle delivery of chemotherapy for enhanced selective cellular uptake and transportation within tumor. Biomacromolecules.

[140] Quílez-Alburquerque J., Saad M.A., Descalzo A.B., Orellana G., Hasan T. (2023 Mar). Hyaluronic acid-poly(lactic-co-glycolic acid) nanoparticles with a ruthenium photosensitizer cargo for photokilling of oral cancer cells. J. Photochem. Photobiol. Chem..

[141] Wang H., Wu J., Williams G.R., Fan Q., Niu S., Wu J. (2019 Dec 13). Platelet-membrane-biomimetic nanoparticles for targeted antitumor drug delivery. J Nanobiotechnology.

[142] Zhou H., Fan Z., Deng J., Lemons P.K., Arhontoulis D.C., Bowne W.B. (2016 May 11). Hyaluronidase embedded in nanocarrier PEG shell for enhanced tumor penetration and highly efficient antitumor efficacy. Nano Lett..

[143] Long L., Xiong W., Lin F., Hou J., Chen G., Peng T. (2023 May 10). Regulating lactate-related immunometabolism and EMT reversal for colorectal cancer liver metastases using shikonin targeted delivery. J. Exp. Clin. Cancer Res..

[144] Guo M., Ling J., Xu X., Ouyang X. (2023 Apr 6). Delivery of doxorubicin by ferric ion-modified mesoporous polydopamine nanoparticles and anticancer activity against HCT-116 cells in vitro. Int. J. Mol. Sci..

[145] Feng L., Chen M., Li R., Zhou L., Wang C., Ye P. (2022 Jan). Biodegradable oxygen-producing manganese-chelated metal organic frameworks for tumor-targeted synergistic chemo/photothermal/photodynamic therapy. Acta Biomater..

[146] Yang Y., Wu S., Zhang Q., Chen Z., Wang C., Jiang S. (2023 Jan 22). A multi-responsive targeting drug delivery system for combination photothermal/chemotherapy of tumor. J. Biomater. Sci. Polym. Ed..

[147] Costa F.J.P., Nave M., Lima-Sousa R., Alves C.G., Melo B.L., Correia I.J. (2023 Mar). Development of Thiol-Maleimide hydrogels incorporating graphene-based nanomaterials for cancer chemo-photothermal therapy. Int J Pharm.

[148] Wang Z., Liu M., Hu J., Wei W., Chen F., Sun B. (2023). A multimodal therapeutic nano-prodrug as an oxidative stress amplifier induces apoptosis and ferroptosis for cancer therapy. New J. Chem..

[149] Liang J., Sun Y., Wang K., Zhang Y., Guo L., Bao Z. (2023 Apr 12). Prussian blue-derived nanoplatform for in situ amplified photothermal/chemodynamic/starvation therapy. ACS Appl. Mater. Interfaces.

[150] Li L., Li J., Hu R., Zhang X., Ding L., Ren G. (2023 Jun 14). Tumor cell targeting and responsive nanoplatform for multimodal-imaging guided chemodynamic/photodynamic/photothermal therapy toward triple negative breast cancer. ACS Appl. Mater. Interfaces.

[151] Lv Z., He S., Wang Y., Zhu X. (2021 Mar 20). Noble metal nanomaterials for NIR‐triggered photothermal therapy in cancer. Adv Healthc Mater.

[152] Zhou Z., Song J., Nie L., Chen X. (2016). Reactive oxygen species generating systems meeting challenges of photodynamic cancer therapy. Chem. Soc. Rev..

[153] Peng F., Zhao F., Shan L., Li R., Jiang S., Zhang P. (2021 Feb). Black phosphorus nanosheets-based platform for targeted chemo-photothermal synergistic cancer therapy. Colloids Surfaces B Biointerfaces.

[154] Maghsoudnia N., Baradaran Eftekhari R., Naderi Sohi A., Norouzi P., Akbari H., Ghahremani M.H. (2020 Sep 13). Mitochondrial delivery of microRNA mimic let-7b to NSCLC cells by PAMAM-based nanoparticles. J. Drug Target..

[155] Maghsoudnia N., Eftekhari R.B., Sohi A.N., Dorkoosh F.A. (2021 Feb 4). Chloroquine assisted delivery of microRNA mimic let-7b to NSCLC cell line by PAMAM (G5) - HA nano-carrier. Curr. Drug Deliv..

[156] Hou L., Chen D., Hao L., Tian C., Yan Y., Zhu L. (2019). Transformable nanoparticles triggered by cancer-associated fibroblasts for improving drug permeability and efficacy in desmoplastic tumors. Nanoscale.

[157] Zhang X., Pan J., Yao M., Palmerston Mendes L., Sarisozen C., Mao S. (2020 Sep). Charge reversible hyaluronic acid-modified dendrimer-based nanoparticles for siMDR-1 and doxorubicin co-delivery. Eur. J. Pharm. Biopharm..

[158] Guo X.L., Kang X.X., Wang Y.Q., Zhang X.J., Li C.J., Liu Y. (2019 Jan). Co-delivery of cisplatin and doxorubicin by covalently conjugating with polyamidoamine dendrimer for enhanced synergistic cancer therapy. Acta Biomater..

[159] Urbiola K., Sanmartín C., Blanco-Fernández L., Tros de Ilarduya C. (2014 Dec). Efficient targeted gene delivery by a novel PAMAM/DNA dendriplex coated with hyaluronic acid. Nanomedicine..

[160] Yang F., Zheng Z., Xue X., Zheng L., Qin J., Li H. (2019 Jan). Targeted eradication of gastric cancer stem cells by CD44 targeting USP22 small interfering RNA-loaded nanoliposomes. Futur Oncol.

[161] Jose G., Lu Y.J., Chen H.A., Hsu H.L., Hung J.T., Anilkumar T.S. (2019 Mar). Hyaluronic acid modified bubble-generating magnetic liposomes for targeted delivery of doxorubicin. J. Magn. Magn Mater..

[162] Anilkumar T.S., Lu Y.J., Chen H.A., Hsu H.L., Jose G., Chen J.P. (2019 Mar). Dual targeted magnetic photosensitive liposomes for photothermal/photodynamic tumor therapy. J. Magn. Magn Mater..

[163] Bai X., Kong M., Wu X., Feng C., Park H., Chen X. (2018). A multi-responsive biomimetic nano-complex platform for enhanced gene delivery. J. Mater. Chem. B.

[164] Li L., Yang S., Song L., Zeng Y., He T., Wang N. (2018). An endogenous vaccine based on fluorophores and multivalent immunoadjuvants regulates tumor micro-environment for synergistic photothermal and immunotherapy. Theranostics.

[165] Liu G.X., Fang G.Q., Xu W. (2014 Aug 29). Dual targeting biomimetic liposomes for paclitaxel/DNA combination cancer treatment. Int. J. Mol. Sci..

[166] Kong M., Hou L., Wang J., Feng C., Liu Y., Cheng X. (2015). Enhanced transdermal lymphatic drug delivery of hyaluronic acid modified transfersomes for tumor metastasis therapy. Chem Commun..

[167] Chen X., Lee S.K., Song M., Zhang T., Han M.S., Chen Y.T. (2021 Dec). RHAMMB-mediated bifunctional nanotherapy targeting Bcl-xL and mitochondria for pancreatic neuroendocrine tumor treatment. Mol Ther - Oncolytics.

[168] Chen S., Lei Q., Qiu W.X., Liu L.H., Zheng D.W., Fan J.X. (2017 Feb). Mitochondria-targeting “Nanoheater” for enhanced photothermal/chemo-therapy. Biomaterials.

[169] Liu J., Wu X., Zhang Y., Liu Y. (2018 Dec 31). Photocleavable supramolecular polysaccharide nanoparticles for targeted drug release in cancer cells. Asian J Org Chem.

[170] Elamin K.M., Yamashita Y., Higashi T., Motoyama K., Arima H. (2018). Supramolecular complex of methyl-β-cyclodextrin with adamantane-grafted hyaluronic acid as a novel antitumor agent. Chem Pharm Bull.

[171] Zhang Y., Yang D., Chen H., Lim W.Q., Phua F.S.Z., An G. (2018 May). Reduction-sensitive fluorescence enhanced polymeric prodrug nanoparticles for combinational photothermal-chemotherapy. Biomaterials.

[172] Sharker S.M., Kim S.M., Kim S.H., In I, Lee H., Park S.Y. (2015). Target delivery of β-cyclodextrin/paclitaxel complexed fluorescent carbon nanoparticles: externally NIR light and internally pH sensitive-mediated release of paclitaxel with bio-imaging. J. Mater. Chem. B.

[173] Chen X., Liu Z., Parker S.G., Zhang X., Gooding J.J., Ru Y. (2016 Jun 29). Light-induced hydrogel based on tumor-targeting mesoporous silica nanoparticles as a theranostic platform for sustained cancer treatment. ACS Appl. Mater. Interfaces.

[174] Guo H., Yi S., Feng K., Xia Y., Qu X., Wan F. (2021 Jan). In situ formation of metal organic framework onto gold nanorods/mesoporous silica with functional integration for targeted theranostics. Chem Eng J.

[175] Zarkesh K., Heidari R., Iranpour P., Azarpira N., Ahmadi F., Mohammadi-Samani S. (2022 Nov). Theranostic hyaluronan coated EDTA modified magnetic mesoporous silica nanoparticles for targeted delivery of cisplatin. J. Drug Deliv. Sci. Technol..

[176] Ghalehkhondabi V., Fazlali A., Soleymani M. (2023 Dec). Preparation of hyaluronic acid-decorated hollow meso-organosilica/poly(methacrylic acid) nanospheres with redox/pH dual responsivity for delivery of curcumin to breast cancer cells. Mater. Today Chem..

[177] Yang D., Wang T., Su Z., Xue L., Mo R., Zhang C. (2016 Aug 31). Reversing cancer multidrug resistance in xenograft models via orchestrating multiple actions of functional mesoporous silica nanoparticles. ACS Appl. Mater. Interfaces.

[178] Zhao Q., Wang S., Yang Y., Li X., Di D., Zhang C. (2017 Sep). Hyaluronic acid and carbon dots-gated hollow mesoporous silica for redox and enzyme-triggered targeted drug delivery and bioimaging. Mater Sci Eng C.

[179] Joseph M.M., Ramya A.N., Vijayan V.M., Nair J.B., Bastian B.T., Pillai R.K. (2020 Sep 14). Targeted theranostic nano vehicle endorsed with self‐destruction and immunostimulatory features to circumvent drug resistance and wipe‐out tumor reinitiating cancer stem cells. Small.

[180] Huang Y.Y., Lee Z.H., Chang K.C., Wu Z.Y., Lee C.C., Tsou M.H. (2023). Mesoporous silica nanoparticles with dual-targeting agricultural sources for enhanced cancer treatment via tritherapy. RSC Adv..

[181] Chen L., Zhou X., Nie W., Zhang Q., Wang W., Zhang Y. (2016 Dec 14). Multifunctional redox-responsive mesoporous silica nanoparticles for efficient targeting drug delivery and magnetic resonance imaging. ACS Appl. Mater. Interfaces.

[182] Hu Y., Yang J., Wei P., Li J., Ding L., Zhang G. (2015). Facile synthesis of hyaluronic acid-modified Fe 3 O 4/Au composite nanoparticles for targeted dual mode MR/CT imaging of tumors. J. Mater. Chem. B.

[183] Kong L., Mu Z., Yu Y., Zhang L., Hu J. (2016). Polyethyleneimine-stabilized hydroxyapatite nanoparticles modified with hyaluronic acid for targeted drug delivery. RSC Adv..

[184] Li J., He Y., Sun W., Luo Y., Cai H., Pan Y. (2014 Apr). Hyaluronic acid-modified hydrothermally synthesized iron oxide nanoparticles for targeted tumor MR imaging. Biomaterials.

[185] Rudiman R., Rahmi N.A. (2023 Sep 27). Latent dirichlet allocation utilization as a text mining method to elaborate learning effectiveness. JSE J Sci Eng.

[186] Devanabanda B, Kasi A (2024). Oxaliplatin. [Updated 2023 May 16]. In: StatPearls [Internet]. Treasure Island (FL).

[187] Zhang C., Liu X., Jin S., Chen Y., Guo R. (2022 Dec 12). Ferroptosis in cancer therapy: a novel approach to reversing drug resistance. Mol. Cancer.

[188] Spill F., Reynolds D.S., Kamm R.D., Zaman M.H. (2016 Aug). Impact of the physical microenvironment on tumor progression and metastasis. Curr. Opin. Biotechnol..

[189] Akhtar M., Haider A., Rashid S., Al-Nabet A.D.M.H. (2019 Jan). Paget's “seed and soil” theory of cancer metastasis: an idea whose time has come. Adv. Anat. Pathol..

[190] Anguille S., Van Acker H.H., Van den Bergh J., Willemen Y., Goossens H., Van Tendeloo V.F., Lapteva N. (2015 May 7). Interleukin-15 dendritic cells harness NK cell cytotoxic effector function in a contact- and IL-15-dependent manner. PLoS One.

[191] Kuai X., Zhu Y., Yuan Z., Wang S., Lin L., Ye X. (2022 Feb). Perfluorooctyl bromide nanoemulsions holding MnO2 nanoparticles with dual-modality imaging and glutathione depletion enhanced HIFU-eliciting tumor immunogenic cell death. Acta Pharm. Sin. B.

[192] Chin Y.T., He Z.R., Chen C.L., Chu H.C., Ho Y., Su P.Y. (2019 Mar 12). Tetrac and NDAT induce anti-proliferation via integrin αvβ3 in colorectal cancers with different K-RAS status. Front. Endocrinol..

[193] Pignatelli P., Umme S., D'Antonio D.L., Piattelli A., Curia M.C. (2023 May 18). Reactive oxygen species produced by 5-aminolevulinic acid photodynamic therapy in the treatment of cancer. Int. J. Mol. Sci..

[194] Namikawa T. (2015). Clinical applications of 5-aminolevulinic acid-mediated fluorescence for gastric cancer. World J. Gastroenterol..

[195] Chiang C.S., Shih I.J., Shueng P.W., Kao M., Zhang L.W., Chen S.F. (2021 Apr). Tumor cell-targeting radiotherapy in the treatment of glioblastoma multiforme using linear accelerators. Acta Biomater..

[196] Wang Y., Yang M., Qian J., Xu W., Wang J., Hou G. (2019 Jan). Sequentially self-assembled polysaccharide-based nanocomplexes for combined chemotherapy and photodynamic therapy of breast cancer. Carbohydr. Polym..

[197] Xu W., Qian J., Hou G., Wang Y., Wang J., Sun T. (2019 Jan). A dual-targeted hyaluronic acid-gold nanorod platform with triple-stimuli responsiveness for photodynamic/photothermal therapy of breast cancer. Acta Biomater..

[198] Dai X., Dong X., Liu Z., Liu G., Liu Y. (2020 Dec 14). Controllable singlet oxygen generation in water based on cyclodextrin secondary assembly for targeted photodynamic therapy. Biomacromolecules.

[199] Niu X., Liu Y., Li X., Wang W., Yuan Z. (2020 Dec 22). NIR light‐driven Bi 2 Se 3 ‐based nanoreactor with “three in one” hemin‐assisted cascade catalysis for synergetic cancer therapy. Adv. Funct. Mater..

[200] Dai X., Zhang B., Zhou W., Liu Y. (2020 Dec 14). High-efficiency synergistic effect of supramolecular nanoparticles based on cyclodextrin prodrug on cancer therapy. Biomacromolecules.

[201] Jarzebska N.T., Mellett M., Frei J., Kündig T.M., Pascolo S. (2021 Jun 14). Protamine-based strategies for RNA transfection. Pharmaceutics.

[202] Seitz B., Baktanian E., Gordon E.M., Anderson W.F., LaBree L., McDonnell P.J. (1998 Jul 20). Retroviral vector-mediated gene transfer into keratocytes: in vitro effects of polybrene and protamine sulfate. Graefe’s Arch. Clin. Exp. Ophthalmol..

[203] Xu X., Li L., Li X., Tao D., Zhang P., Gong J. (2020 Nov). Aptamer-protamine-siRNA nanoparticles in targeted therapy of ErbB3 positive breast cancer cells. Int J Pharm.

[204] Aziz M., Garduno R., Mirani Z.A., Baqai R., Sheikh A.S., Nazir H. (2019 Jul). Determination of antimicrobial effect of protamine by transmission electron microscopy and SDS PAGE on Pseudomonas aeruginosa isolates from diabetic foot infection. Iran J Basic Med Sci.

[205] Priya S.S., Rekha M.R., Sharma C.P. (2014 Feb). Pullulan–protamine as efficient haemocompatible gene delivery vector: synthesis and in vitro characterization. Carbohydr. Polym..

[206] Fukushige K., Tagami T., Naito M., Goto E., Hirai S., Hatayama N. (2020 Jun). Developing spray-freeze-dried particles containing a hyaluronic acid-coated liposome–protamine–DNA complex for pulmonary inhalation. Int J Pharm.

[207] Manju C.A., Jeena K., Ramachandran R., Manohar M., Ambily A.M., Sajesh K.M. (2021 Jan 1). Intracranially injectable multi-siRNA nanomedicine for the inhibition of glioma stem cells. Neuro-Oncology Adv.

[208] Yang J., Zhao R., Feng Q., Zhuo X., Wang R. (2021 Feb 13). Development of a carrier system containing hyaluronic acid and protamine for siRNA delivery in the treatment of melanoma. Invest New Drugs.

[209] Cui X., Deng X., Liang Z., Lu J., Shao L., Wang X. (2021). Multicomponent-assembled nanodiamond hybrids for targeted and imaging guided triple-negative breast cancer therapy via a ternary collaborative strategy. Biomater. Sci..

[210] De Groot C. (2001 Jun 1). In vitro biocompatibility of biodegradable dextran-based hydrogels tested with human fibroblasts. Biomaterials.

[211] Petrovici A.R., Pinteala M., Simionescu N. (2023 Jan 21). Dextran formulations as effective delivery systems of therapeutic agents. Molecules.

[212] Chen B., Wu Y., Wu H., Meng X., Chen H. (2023 Feb 27). Establishment of food allergy model in dextran sulfate sodium induced colitis mice. Foods.

[213] Ding C., Shi Z., Ou M., Li Y., Huang L., Wang W. (2023 Nov). Dextran-based micelles for combinational chemo-photodynamic therapy of tumors via in vivo chemiluminescence. Carbohydr. Polym..

[214] Wang H., Han X., Dong Z., Xu J., Wang J., Liu Z. (2019 Jul 22). Hyaluronidase with pH‐responsive dextran modification as an adjuvant nanomedicine for enhanced photodynamic‐immunotherapy of cancer. Adv. Funct. Mater..

[215] Unterweger H., Tietze R., Janko C., Zaloga J., Lyer S., Taccardi N. (2014 Aug). Development and characterization of magnetic iron oxide nanoparticles with a cisplatin-bearing polymer coating for targeted drug delivery. Int J Nanomedicine.

[216] Wilbur D.S., Hamlin D.K., Chyan M.K., Brechbiel M.W. (2008 Jan 1). Streptavidin in antibody pretargeting. 5. Chemical modification of recombinant streptavidin for labeling with the α-particle-emitting radionuclides 213 Bi and 211 at. Bioconjug Chem.

[217] Jain A., Cheng K. (2017 Jan). The principles and applications of avidin-based nanoparticles in drug delivery and diagnosis. J Control Release.

[218] Beals N., Kasibhatla N., Basu S. (2019 Feb 18). Efficient delivery of plasmid DNA using incorporated nucleotides for precise conjugation of targeted nanoparticles. ACS Appl. Bio Mater..

[219] Zhang M., Xu C., Wen L., Han M.K., Xiao B., Zhou J. (2016 Dec 15). A hyaluronidase-responsive nanoparticle-based drug delivery system for targeting colon cancer cells. Cancer Res..

[220] Xu W., Qian J., Hou G., Suo A., Wang Y., Wang J. (2017 Oct 25). Hyaluronic acid-functionalized gold nanorods with pH/NIR dual-responsive drug release for synergetic targeted photothermal chemotherapy of breast cancer. ACS Appl. Mater. Interfaces.

[221] Xu W., Wang J., Qian J., Hou G., Wang Y., Ji L. (2019 Oct). NIR/pH dual-responsive polysaccharide-encapsulated gold nanorods for enhanced chemo-photothermal therapy of breast cancer. Mater Sci Eng C.

[222] Pornpitchanarong C., Rojanarata T., Opanasopit P., Ngawhirunpat T., Patrojanasophon P. (2020 Dec). Catechol-modified chitosan/hyaluronic acid nanoparticles as a new avenue for local delivery of doxorubicin to oral cancer cells. Colloids Surfaces B Biointerfaces.

[223] Choi C.A., Lee J.E., Mazrad Z.A.I., In I., Jeong J.H., Park S.Y. (2018 Jul). Redox- and pH-responsive fluorescent carbon nanoparticles-MnO2-based FRET system for tumor-targeted drug delivery in vivo and in vitro. J. Ind. Eng. Chem..

[224] Li J., Hu Y., Yang J., Wei P., Sun W., Shen M. (2015 Jan). Hyaluronic acid-modified Fe3O4@Au core/shell nanostars for multimodal imaging and photothermal therapy of tumors. Biomaterials.

[225] Li R.T., Chen M., Yang Z.C., Chen Y.J., Huang N.H., Chen W.H. (2022). AIE-based gold nanostar-berberine dimer nanocomposites for PDT and PTT combination therapy toward breast cancer. Nanoscale.

[226] Gui W., Zhang J., Chen X., Yu D., Ma Q. (2018 Jan 18). N-Doped graphene quantum dot@mesoporous silica nanoparticles modified with hyaluronic acid for fluorescent imaging of tumor cells and drug delivery. Microchim. Acta.

[227] Chang Z., Yang Y., Zhao B., Li H., Guan Y., Zhao Y. (2022 Aug). Dual-targeting magnetic fluorescent mesoporous organosilicon hollow nanospheres for gambogic acid loading, sustained release and anti-tumor properties. J. Mol. Liq..

[228] Fang J., Liu Y., Chen Y., Ouyang D., Yang G., Yu T. (2018 Oct). Graphene quantum dots-gated hollow mesoporous carbon nanoplatform for targeting drug delivery and synergistic chemo-photothermal therapy. Int J Nanomedicine.

[229] Ziaee N., Farhadian N., Abnous K., Matin M.M., Khoshnood A., Yaghoobi E. (2023 Aug). Dual targeting of Mg/N doped-carbon quantum dots with folic and hyaluronic acid for targeted drug delivery and cell imaging. Biomed. Pharmacother..

[230] Tan H., Liu Y., Hou N., Cui S., Liu B., Fan S. (2022 Mar). Tumor microenvironment pH-responsive pentagonal gold prism-based nanoplatform for multimodal imaging and combined therapy of castration-resistant prostate cancer. Acta Biomater..

[231] Bin Li, Chen T., Peña J., Xing J., Zeng L. (2020 Apr 20). Hyaluronic acid-modified fluorescent probe for dual color imaging of living cell. ACS Appl. Bio Mater..

[232] Yang H., Miao Y., Chen L., Li Z., Yang R., Xu X. (2020 Apr). Redox-responsive nanoparticles from disulfide bond-linked poly-(N-ε-carbobenzyloxy-l-lysine)-grafted hyaluronan copolymers as theranostic nanoparticles for tumor-targeted MRI and chemotherapy. Int. J. Biol. Macromol..

[233] Luo Y., Li Y., Li J., Fu C., Yu X., Wu L. (2019). Hyaluronic acid-mediated multifunctional iron oxide-based MRI nanoprobes for dynamic monitoring of pancreatic cancer. RSC Adv..

[234] Kim H.M., Kim D.M., Jeong C., Park S.Y., Cha M.G., Ha Y. (2018 Sep 17). Assembly of plasmonic and magnetic nanoparticles with fluorescent silica shell layer for tri-functional SERS-magnetic-fluorescence probes and its bioapplications. Sci. Rep..

[235] Wang Y., Zhang W., Sun P., Cai Y., Xu W., Fan Q. (2019). A novel multimodal NIR-II nanoprobe for the detection of metastatic lymph nodes and targeting chemo-photothermal therapy in oral squamous cell carcinoma. Theranostics.

[236] Liang X., Fang L., Li X., Zhang X., Wang F. (2017 Jul). Activatable near infrared dye conjugated hyaluronic acid based nanoparticles as a targeted theranostic agent for enhanced fluorescence/CT/photoacoustic imaging guided photothermal therapy. Biomaterials.

[237] Li Y., Zhao L., Li X.F. (2021 Jan 1). Hypoxia and the tumor microenvironment. Technol. Cancer Res. Treat..

[238] Ziello J.E., Jovin I.S., Huang Y. (2007 Jun). Hypoxia-Inducible Factor (HIF)-1 regulatory pathway and its potential for therapeutic intervention in malignancy and ischemia. Yale J. Biol. Med..

[239] Zimna A., Kurpisz M. (2015). Hypoxia-inducible factor-1 in physiological and pathophysiological angiogenesis: applications and therapies. BioMed Res. Int..

[240] Infantino V., Santarsiero A., Convertini P., Todisco S., Iacobazzi V. (2021 May 27). Cancer cell metabolism in hypoxia: role of HIF-1 as key regulator and therapeutic target. Int. J. Mol. Sci..

[241] Yang K., Liao Z., Wu Y., Li M., Guo T., Lin J., Ramkumar V. (2020 Dec 28). Curcumin and glu-GNPs induce radiosensitivity against breast cancer stem-like cells. BioMed Res. Int..

[242] Han L., Wang Y., Huang X., Liu F., Ma C., Feng F. (2020 Oct). Specific-oxygen-supply functionalized core-shell nanoparticles for smart mutual-promotion between photodynamic therapy and gambogic acid-induced chemotherapy. Biomaterials.

[243] Yang H.Y., Jang M.S., Sun X.S., Liu C.L., Lee J.H., Li Y. (2023 Aug). CD44-mediated tumor homing of hyaluronic acid nanogels for hypoxia-activated photodynamic therapy against tumor. Colloids Surfaces B Biointerfaces.

[244] Tseng S.J., Kempson I.M., Huang K.Y., Li H.J., Fa Y.C., Ho Y.C. (2018 Oct 23). Targeting tumor microenvironment by bioreduction-activated nanoparticles for light-triggered virotherapy. ACS Nano.

[245] Wang Y., Zou L., Qiang Z., Jiang J., Zhu Z., Ren J. (2020 Jun 8). Enhancing targeted cancer treatment by combining hyperthermia and radiotherapy using Mn–Zn ferrite magnetic nanoparticles. ACS Biomater. Sci. Eng..

[246] Xu H., Su Z., Zhang H., Zhang Y., Bao Y., Zhang H. (2023 Jun). Cu2+-pyropheophorbide-a-cystine conjugate-mediated multifunctional mesoporous silica nanoparticles for photo-chemodynamic therapy/GSH depletion combined with immunotherapy cancer. Int J Pharm.

[247] Chen K., Sun X., Liu Y., Yang Y., Shi M., Yu J. (2022 Oct 17). CeO 2 -decorated metal–organic framework for enhanced photodynamic therapy. Inorg. Chem..

[248] Cai X., Luo Y., Zhang W., Du D., Lin Y. (2016 Aug 31). pH-sensitive ZnO quantum dots–doxorubicin nanoparticles for lung cancer targeted drug delivery. ACS Appl. Mater. Interfaces.

[249] Liu Y., Qiao L., Zhang S., Wan G., Chen B., Zhou P. (2018 Jan). Dual pH-responsive multifunctional nanoparticles for targeted treatment of breast cancer by combining immunotherapy and chemotherapy. Acta Biomater..

[250] Pandey A., Singh K., Patel S., Singh R., Patel K., Sawant K. (2019 May). Hyaluronic acid tethered pH-responsive alloy-drug nanoconjugates for multimodal therapy of glioblastoma: an intranasal route approach. Mater Sci Eng C.

[251] Liu H.N., Guo N.N., Wang T.T., Guo W.W., Lin M.T., Huang-Fu M.Y. (2018 Mar 5). Mitochondrial targeted doxorubicin-triphenylphosphonium delivered by hyaluronic acid modified and pH responsive nanocarriers to breast tumor: in vitro and in vivo studies. Mol. Pharm..

[252] Wang X., Song Y., Yu L., Xue X., Pang M., Li Y. (2023 Jul 26). Co-delivery of hesperetin and cisplatin via hyaluronic acid-modified liposome for targeted inhibition of aggression and metastasis of triple-negative breast cancer. ACS Appl. Mater. Interfaces.

[253] Zeng J., Sun P., Fang X., Jiang Y., Wu Z., Qi X. (2023 Mar). “Shell-Core” bilayer nanoparticle as chemotherapeutic drug Co-delivery platforms render synchronized microenvironment respond and enhanced antitumor effects. Int J Nanomedicine.

[254] Chen J., Tan X., Huang Y., Xu C., Zeng Z., Shan T. (2022 May). Reactive oxygen species-activated self-amplifying prodrug nanoagent for tumor-specific Cu-chelate chemotherapy and cascaded photodynamic therapy. Biomaterials.

[255] Carovac A., Smajlovic F., Junuzovic D. (2011). Application of ultrasound in medicine. Acta Inform Medica.

[256] Bor R., Fábián A., Szepes Z. (2016). Role of ultrasound in colorectal diseases. World J. Gastroenterol..

[257] Wolverson M.K., Nouri S., Joist J.H., Sundaram M., Heiberg E. (1981 Aug). The direct visualization of blood flow by real-time ultrasound: clinical observations and underlying mechanisms. Radiology.

[258] Shiva M., Wei C., Molana H., Nabi G. (2022 Jan 28). Cost-effectiveness of prostate cancer detection in biopsy-naïve men: ultrasound shear wave elastography vs. Multiparametric diagnostic magnetic resonance imaging. Healthcare.

[259] Zhu L., Yang Y., Li X., Zheng Y., Li Z., Chen H. (2022 Dec). Facile preparation of indocyanine green and tiny gold nanoclusters co-loaded nanocapsules for targeted synergistic sono-/photo-therapy. J. Colloid Interface Sci..

[260] Ben Daya S.M., Paul V., Awad N.S., Al Sawaftah N.M., Al Sayah M.H., Husseini G.A. (2021 Jan 1). Targeting breast cancer using hyaluronic acid-conjugated liposomes triggered with ultrasound. J. Biomed. Nanotechnol..

[261] Feng Q., Zhang W., Yang X., Li Y., Hao Y., Zhang H. (2018 Mar 15). pH/ultrasound dual‐responsive gas generator for ultrasound imaging‐guided therapeutic inertial cavitation and sonodynamic therapy. Adv Healthc Mater.

[262] Zhao H., Wu M., Zhu L., Tian Y., Wu M., Li Y. (2018). Cell-penetrating peptide-modified targeted drug-loaded phase-transformation lipid nanoparticles combined with low-intensity focused ultrasound for precision theranostics against hepatocellular carcinoma. Theranostics.

[263] Shakeri M., Delavari H.H., Montazerabadi A., Yourdkhani A. (2022 Sep). Hyaluronic acid-coated ultrasmall BiOI nanoparticles as a potentially targeted contrast agent for X-ray computed tomography. Int. J. Biol. Macromol..

[264] Xu X., Chong Y., Liu X., Fu H., Yu C., Huang J. (2019 Jan). Multifunctional nanotheranostic gold nanocages for photoacoustic imaging guided radio/photodynamic/photothermal synergistic therapy. Acta Biomater..

[265] Cheng Y., Lu T., Wang Y., Song Y., Wang S., Lu Q. (2019 Aug 5). Glutathione-mediated clearable nanoparticles based on ultrasmall Gd 2 O 3 for MSOT/CT/MR imaging guided photothermal/radio combination cancer therapy. Mol. Pharm..

[266] Song Y., Wang Y., Zhu Y., Cheng Y., Wang Y., Wang S. (2019 Aug 10). Biomodal tumor‐targeted and redox‐responsive Bi 2 Se 3 hollow nanocubes for MSOT/CT imaging guided synergistic low‐temperature photothermal radiotherapy. Adv Healthc Mater.

[267] Song Y., Wang J., Liu L., Sun Q., You Q., Cheng Y. (2018 May 7). One-pot synthesis of a bismuth selenide hexagon nanodish complex for multimodal imaging-guided combined antitumor phototherapy. Mol. Pharm..

[268] Guo Z., Zheng K., Tan Z., Liu Y., Zhao Z., Zhu G. (2018). Overcoming drug resistance with functional mesoporous titanium dioxide nanoparticles combining targeting, drug delivery and photodynamic therapy. J. Mater. Chem. B.

[269] Jin Y., Ma X., Feng S., Liang X., Dai Z., Tian J. (2015 Dec 16). Hyaluronic acid modified tantalum oxide nanoparticles conjugating doxorubicin for targeted cancer theranostics. Bioconjug Chem..

[270] Yang Y., Yue S., Qiao Y., Zhang P., Jiang N., Ning Z. (2021 Apr 12). Activable multi-modal nanoprobes for imaging diagnosis and therapy of tumors. Front. Chem..

[271] Patra J.K., Das G., Fraceto L.F., Campos E.V.R., Rodriguez-Torres M. del P., Acosta-Torres L.S. (2018 Dec 19). Nano based drug delivery systems: recent developments and future prospects. J Nanobiotechnology.

[272] Chen L., Huang J., Li X., Huang M., Zeng S., Zheng J. (2022 May 31). Progress of nanomaterials in photodynamic therapy against tumor. Front. Bioeng. Biotechnol..

[273] Lin B., Dai R., Liu Z., Li W., Bai J., Zhang G. (2023 Aug). Dual-targeting lanthanide-ICG-MOF nanoplatform for cancer Theranostics: NIR II luminescence imaging guided sentinel lymph nodes surgical navigation. J. Photochem. Photobiol. B Biol..

[274] Sun X., Xu Y., Guo Q., Wang N., Wu B., Zhu C. (2022 Feb 1). A novel nanoprobe for targeted imaging and photothermal/photodynamic therapy of lung cancer. Langmuir.

[275] NIH (2024).

